# Determination of Optimal Harvest Time in *Cannabis sativa* L. Based upon Stigma Color Transition

**DOI:** 10.3390/plants14101532

**Published:** 2025-05-20

**Authors:** Jonathan Tran, Adam M. Dimech, Simone Vassiliadis, Aaron C. Elkins, Noel O. I. Cogan, Erez Naim-Feil, Simone J. Rochfort

**Affiliations:** 1 Agriculture Victoria Research, Department of Energy, Environment and Climate Action, AgriBio Centre for Agribioscience, Bundoora, Melbourne, VIC 3083, Australia; adam.dimech@agriculture.vic.gov.au (A.M.D.); simone.vassiliadis@agriculture.vic.gov.au (S.V.); aaron.elkins@agriculture.vic.gov.au (A.C.E.); noel.cogan@agriculture.vic.gov.au (N.O.I.C.); erez.naim-feil@agriculture.vic.gov.au (E.N.-F.); simone.rochfort@agriculture.vic.gov.au (S.J.R.); 2 School of Applied Systems Biology, La Trobe University, Bundoora, Melbourne, VIC 3083, Australia

**Keywords:** cannabinoid, CBD, THC, image analysis, RGB, CIELAB, PlantCV, LCMS, NIR, PLS

## Abstract

*Cannabis sativa* L. is cultivated for therapeutic and recreational use. Delta-9 tetrahydrocannabinol (THC) and cannabidiol (CBD) are primarily responsible for its psychoactive and medicinal effects. As the global cannabis industry continues to expand, constant review and optimization of horticultural practices are needed to ensure a reliable harvest and improved crop quality. There is currently uncertainty about the optimal harvest time of *C. sativa*, i.e., when cannabinoid concentrations are at their highest during inflorescence maturation. At present, growers observe the color transition of stigmas from white to amber as an indicator of harvest time. This research investigates the relationship between stigma color and cannabinoid concentration using liquid chromatography–mass spectrometry (LCMS) and digital image analysis. Additionally, early screening prediction models have also been developed for six cannabinoids using near-infrared (NIR) spectroscopy and LCMS to assist in early cannabinoid determination. Among the genotypes grown, 22 of 25 showed cannabinoid concentration peaks between the third (mostly amber) and fourth (fully amber) stages; however, some genotypes peaked within the first (no amber) and second (some amber) stages. We have determined that the current ‘rule of thumb’ of harvesting when a cannabis plant is mostly amber is still a useful approximation in most cases; however, studies on individual genotypes should be performed to determine their individual optimal harvest time based on the desired cannabinoid profile or total cannabinoid concentration.

## 1. Introduction

*Cannabis sativa* L. is an annual herbaceous flowering plant that has been cultivated for several millennia. The first recorded use of cannabis dates back to 2800 BCE in Emperor Shen Nung’s *Pharmacopeia*, where it is claimed *C. sativa* could treat arthritis, depression, inflammation, pain and asthma [1]. Previously, two species of *Cannabis, C. indica* and *C. ruderalis,* were recognized, with each species having different leaf size and plant structure [2,3]. However, recent studies have confirmed that *C. indica* and *C. ruderalis* are subspecies of *C. sativa* [4,5,6].

*C. sativa* contains over 125 identified cannabinoids, which can be divided into two categories: acidic and neutral forms. These are determined by the presence or absence of a carboxyl group. Cannabis is diecious, and it is the inflorescences on the female plants that contain the greatest concentration of cannabinoids. Cannabidiol (CBD) and tetrahydrocannabinol (THC) are the major cannabinoids targeted for their medicinal properties; however they are in low abundance relative to their acidic precursors, cannabidiolic acid (CBDA) and tetrahydrocannabinolic acid (THCA). Decarboxylation by heating is required to facilitate the conversion to the active ingredients. CBD is non-psychoactive and is used in seizure management [7], pain relief [8], anti-anxiety medication [9], and Parkinson’s disease treatment [10]. THC is effective for chronic pain and multiple sclerosis [11], post-traumatic stress disorder (PTSD) [12], and chemotherapy-induced nausea and vomiting [13]. While CBDA and THCA are not typically used medicinally, CBDA has been shown to have anti-anxiety [14] properties, while THCA has been shown to have neuroprotective [15] properties; both of these acidic cannabinoids exhibit anti-nausea and anti-inflammatory properties [16,17,18]. The effects of minor cannabinoids found in *C. sativa* range from various anti-inflammatory, analgesic, neuroprotective, and anti-convulsant to anti-cancer effects [19]. The significant minor cannabinoids of interest are cannabigerolic acid (CBGA), cannabigerol (CBG), cannabichromenic acid (CBCA), cannabichromene (CBC), cannabinolic acid (CBNA), cannabinol (CBN), cannabidivarinic acid (CBDVA), cannabidivarin (CBDV), tetrahydrocannabivarin acid (THCVA), and tetrahydrocannabivarin (THCV).

The total cannabis industry revenue is worth USD 58 billion globally [20]. With increasing demand, it is vital that industry continues to optimize horticultural processes and increase cannabinoid yields. Most cannabis cultivation protocols rely on optimized lighting, climate control, nutrient management, pruning, watering, harvest scheduling, and genetic selection. For instance, Bevan et al. [21] used response surface analysis to optimize nutrient composition for the soilless production of cannabis. Massuela et al. [22] studied harvest time and found that the total CBD concentration peaked at the 9th week of flowering. Massuela also investigated pruning techniques and found that the removal of the apical meristem at the tenth node resulted in a higher yield, followed by plants that were not pruned at all and plants that only had their lateral shoots removed. Linder et al. [23] investigated peak total THC and CBD concentrations (including THCA and CBDA) versus harvest time within two genotypes, ‘Cherry Wine’ and ‘BaOx’, and found that peak THC concentration in ‘Cherry Wine’ was reached on day 63 of flower initiation, whereas ‘BaOx’ was on day 75. For ‘Cherry Wine’, CBD concentration plateaued within the 84-day study period, whereas ‘BaOx’ never plateaued. That study showed that the optimal harvest time varies between genotypes and that peak THC and CBD levels will vary in quantity and will have different peak time points, posing a challenge to find a consistent timeframe that fits all genotypes.

To further optimize the harvesting process of cannabis, early screening techniques for cannabinoid concentration using portable handheld near-infrared spectrometers have been employed to help cultivators in optimizing cultivation and harvest practices [24,25]. Near-infrared (NIR) spectroscopy enables organic bonds to be detected at certain wavelengths within the near-infrared spectrum (750–1400 nm). These NIR spectral data are then paired with liquid chromatography–mass spectrometry (LCMS) data to build prediction models. NIR benefits from being rapid and non-destructive. Although not as accurate as LCMS, the prediction models have well-defined limits to help inform growers on the accuracy and precision of a prediction model. Deidda et al. [25] utilized multiple NIR devices to build prediction models for THC concentration. A recent study by Tran et al. [24] developed prediction models for THCA concentration and produced discriminant models to differentiate between ‘high-THCA’ and ‘even-ratio’ genotypes. These studies confirmed the viability of NIR analysis on entirely intact inflorescences but limited the analysis to one cannabinoid. The current study has expanded upon this work by developing prediction models for CBDA, CBD, CBN, CBNA, and CBDVA.

Traditionally, growers have used the color of stigmas, floral organs that receive pollen, to provide an indication of the likely period of greatest cannabinoid production. Cannabis stigmas initially appear white and then gradually change color to yellow, orange, amber, red, or brown depending on the genotype. Some studies also investigate the change in trichome color development to assess maturation [26,27]. There are many theories amongst laypeople about why there should be a correlation between stigma color and cannabinoid production, but this has not been explored scientifically. The present study seeks to explore whether stigma color change is directly linked to peak cannabinoid concentration and aims to develop accessible and non-invasive NIR and RGB analysis tools to assist growers in determining optimal harvest time.

## 2. Results and Discussion

### 2.1. Calculation of Amber Score

**Figure 1:**
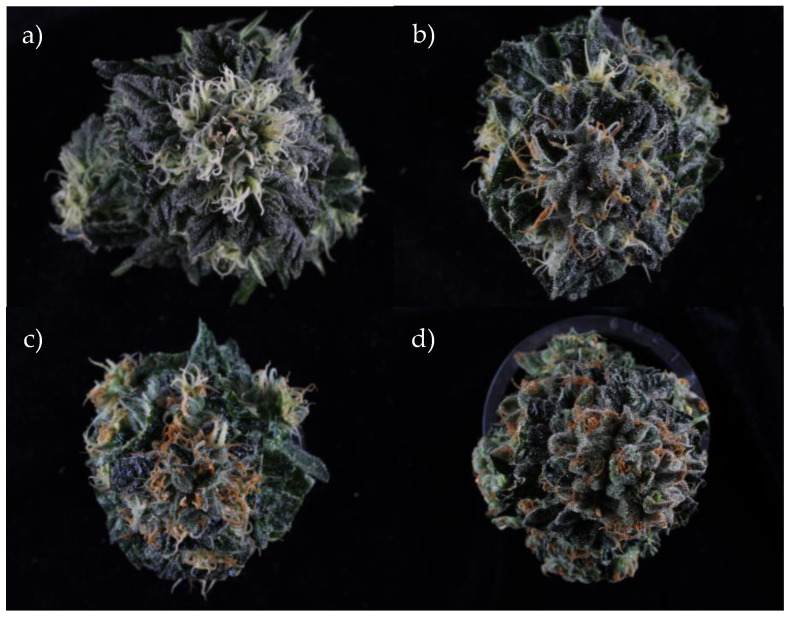
Cannabis inflorescences at each amber stage: (**a**) fully white, (**b**) some amber, (**c**) mostly amber, (**d**) fully amber.

The amber score, which was a measure of the ‘yellowness’ of the stigmas, was visually assessed based on the perceived color of stigmas on a gradient from white to amber on individual inflorescences. The manual amber score was simplified into four stages; stage 1: fully white, stage 2: some amber; stage 3: mostly amber, stage 4: fully amber (Figure 1). These assessments were performed manually, and it was decided by the authors to simplify the categorization into stages to streamline the process and to reduce bias in results. Manual assessments are inherently prone to bias as their decisions may be influenced by an individual’s color perception, previous experience with cannabis harvests, and general user error. Assessment was performed by the same person for all the plants immediately prior to harvest.

Digital image analysis was performed using a customized image analysis pipeline in PlantCV. The automated results were evaluated against the visual data and correlations measured to determine whether image analysis could be used to accurately and quantitatively evaluate the amber score.

**Figure 2:**
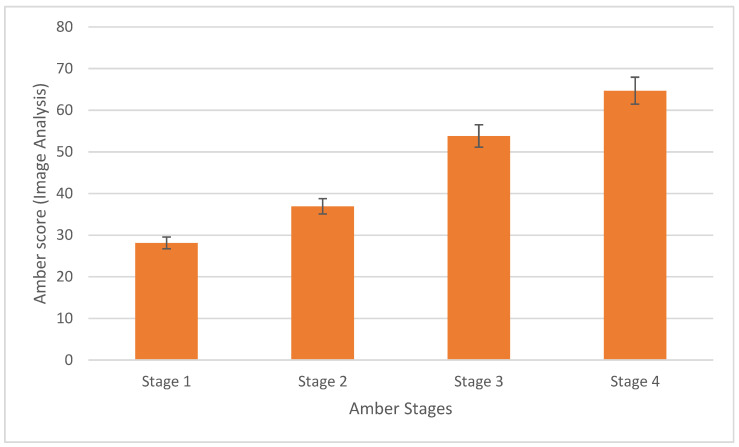
The mean automated amber score calculated using image analysis for each inflorescence, categorized by its manual amber score. Error bars indicate the standard error. (*n* = 25 for each amber stage).

The mean automated amber score was calculated for each inflorescence and was then compared to the manual amber scores that were calculated for the same inflorescence (Figure 2). Stage 1 averaged an amber score of 28, with a standard error of 4.1; stage 2 averaged an amber score of 37, with a standard error of 3.5; stage 3 averaged an amber score of 54, with a standard error of 3.6; and stage 4 averaged an amber score of 65, with a standard error of 3.5. The automated amber score identified an increasing trend from white to amber across the four stages.

**Figure 3:**
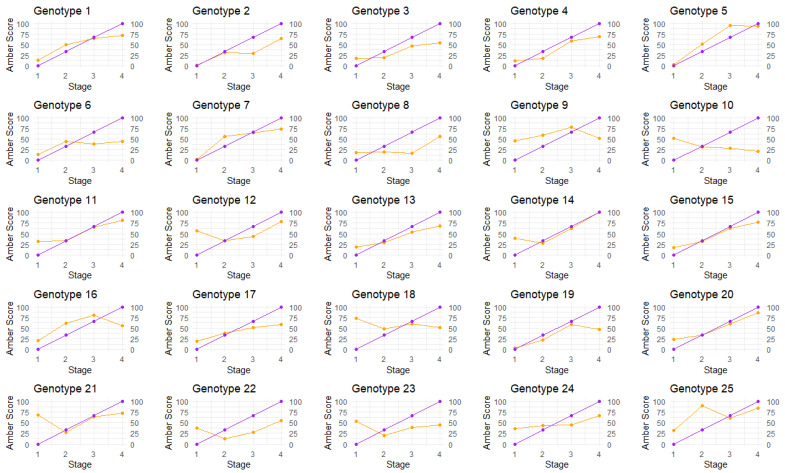
The comparison of mean amber scores using visual versus image analysis in each plant over four amber stages. Purple = manual scoring; orange = automated scoring.

When comparing manual amber scores against automated amber scores (Figure 3), the majority of amber scores show similar trending. There were some exceptions to this, where the automated amber scores declined over time (Figure 3, Genotypes 10 and 18) or were volatile (Figure 3, genotype 25). This was due to the misclassification of stigmas in the image analysis pipeline that arose from suboptimal color thresholding. Correctly identifying and isolating trichomes and stigmas from an inflorescence when both are almost identically colored is difficult. On occasion, more trichomes were included in the analysis than was expected. Although trichomes also experience a color change from white to amber, some may exhibit the color change later than the stigmas; this had an effect of lowering the automated amber score for those plants, as the trichomes remained white longer than the stigmas and lowered the amber score. Generally, stigmas appear larger than trichomes, so for most genotypes, this was not an issue. Potential improvements to the method would be the narrowing of the parameters to ensure the image analysis captures larger structures only, such as stigmas, but to also ensure that it is not capturing other structures such as leaves or other sources of plant biomass. Nevertheless, the methodology used here demonstrates that in the majority of cases, it can be reliably deployed to accurately quantify the color of stigmas in cannabis flowers.

### 2.2. Total Cannabinoid Concentration Versus Amber Score

**Figure 4:**
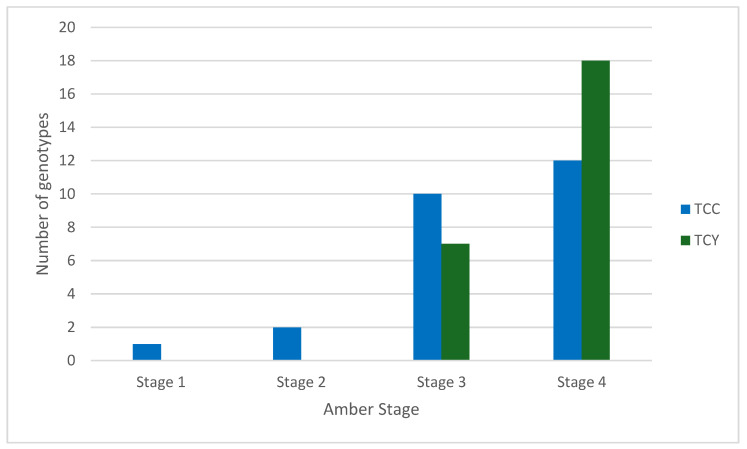
The number of genotypes where the total cannabinoid concentration (TCC) and total cannabinoid yield (TCY) peaked at each amber stage. LCMS data on all cannabinoids analyzed are present in Supplementary Table S1.

Total cannabinoid concentration (TCC) was calculated by adding the LCMS results of 14 cannabinoids: CBDA, THCA, CBD, THC, CBC, CBN, CBDVA, CBDV, CBGA, CBG, THCV, THCVA, CBNA, and CBCA. Samples were collected from four inflorescences taken from each of 25 genotypes, resulting in 100 samples. One inflorescence from each genotype was analyzed for peak TCC for each genotype at each amber stage. TCC ranged from 4.03 mg/g to 87.06 mg/g across all amber stages. A comparison of peak TCC and amber stage (Figure 4) shows that 22 genotypes peaked at stages 3 and 4; of those, 10 genotypes showed the highest concentration in stage 3, and 12 genotypes showed the highest concentration in stage 4. One genotype recorded the maximum TCC in stage 1 and slowly decreased from stage 2 to stage 4. Total cannabinoid yield (TCY) was calculated by multiplying the total cannabinoid concentration (mg/g) against the gross inflorescence weight (g). When looking at TCY, 7 genotypes peaked in stage 3 and 18 genotypes peaked in stage 4; this shows that most genotypes provide higher TCY, which is likely attributed to the growth continuity of the inflorescence and trichome development.

**Figure 5:**
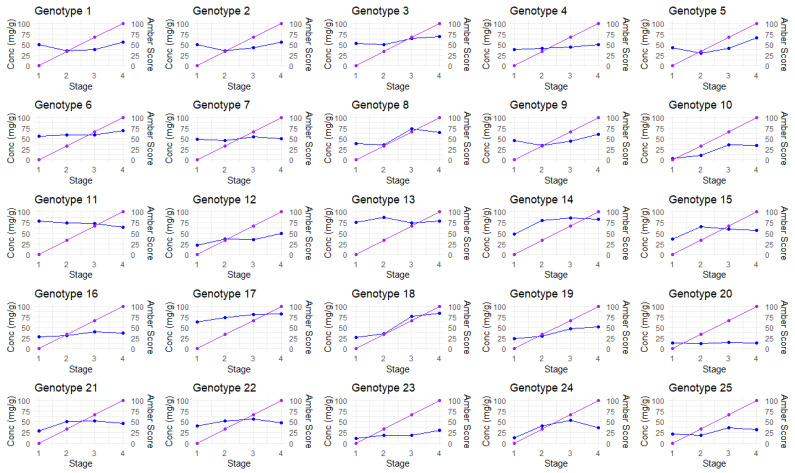
The total cannabinoid concentration (TCC) versus the visual amber score of each plant over the four amber stages. Blue = total cannabinoid concentration (mg/g); purple = manual amber score.

**Figure 6:**
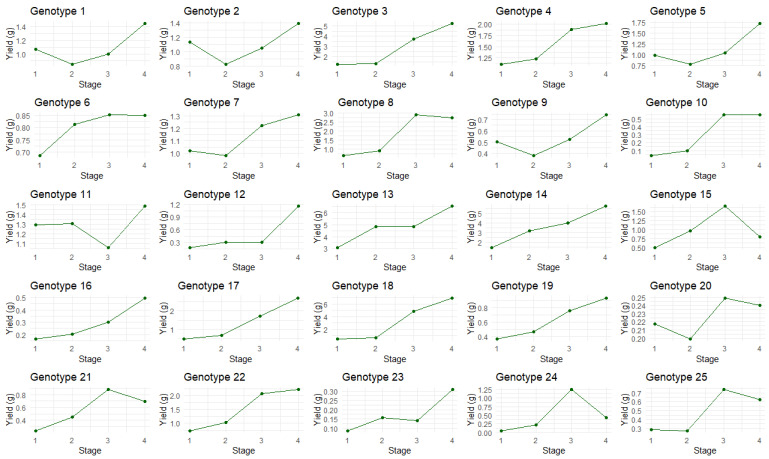
The total cannabinoid yield of each plant over the four amber stages. Dark green: total cannabinoid yield (g).

TCC tended to fluctuate over time (Figure 5), as did TCY (Figure 6). By stage 4, inflorescences were visually inspected (manually) and found to either be completely amber or the plant showed signs of senescence and needed to be harvested. In general, most plants had increased TCC and TCY over time, with the majority peaking or plateauing at stages 3 and 4. However, there were a few exceptions. Genotype 11 produced maximum TCC in stage 1 (Day 31), reaching 78.53 mg/g, with TCC then decreasing to 64.65 mg/g in stage 4, making it a high-concentration plant in Stage 1. Despite genotype 11 having high TCC across all stages, the low inflorescence weight and size means the TCY is relatively low, 1.487 g. The authors suggest that this high TCC and low TCY is due to the plant biomass of genotype 11, which is low compared to other genotypes. It is possible that this genotype prioritizes cannabinoid production over vegetative growth. This characteristic may make it highly desirable for mass cannabinoid production, depending on other physiological and plant architecture traits. However, further studies with more plant (genotype) replicates are needed to confirm the original observations and assess this potentially high-value genotype in more detail. Genotype 13 recorded the highest TCC in stage 2, at 87.06 mg/g, and the second highest cannabinoid yield of 6.56 g in stage 4, making it a high-performing plant in both TCC and TCY. Genotype 15 saw a sharp increase in TCC from the first stage (35.68 mg/g) to stage 2 (65.40 mg/g), where it peaked, and then a decrease in concentration in stages 3 and 4; however, when observing TCY, the peak was observed in the third stage, at 1.65 g.

Genotypes that peaked in TCY within stage 3 could be very beneficial for industry, since earlier harvesting results in faster production. However, growers should evaluate their lines with an adequate number of replicates, because it has been demonstrated that inflorescences harvested at the same time can vary due to microclimatic variations in glasshouse conditions [28]. In some cases, plants showed no significant change in TCC over time. For instance, genotype 20 remained consistent, with a TCC ranging between 12.05 and 15.03 mg/g, despite the amber score increasing. Genotype 20 exhibited low TCY values, ranging from 0.20 to 0.25 g, due to its low inflorescence weight. It did, however, have a peak in TCC at stage 3, which would time its maximum production with the period when harvesting would be expected by industry.

**Figure 7:**
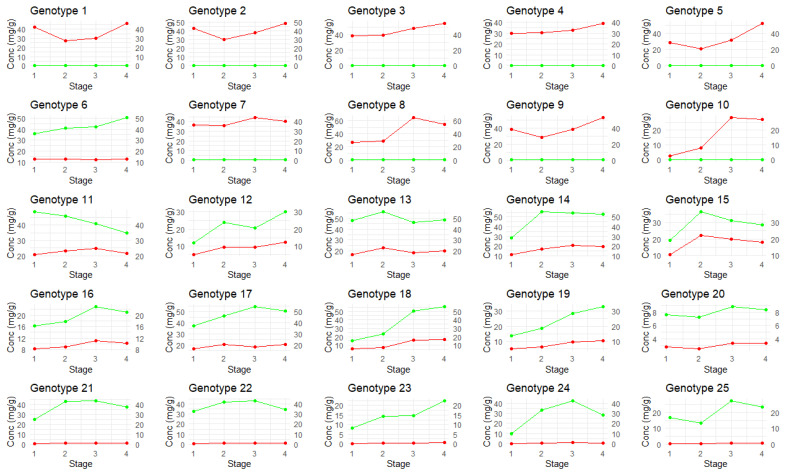
The THCA and CBDA concentrations of each plant over the four amber stages. Red = THCA concentration (mg/g); green = CBDA concentration (mg/g).

The individual cannabinoid concentrations for all 14 cannabinoids at each time point were plotted and are available in Supplementary Figures S1.1–S1.14. The main cannabinoids of interest, CBDA and THCA, are shown in Figure 7. The genotypes were deliberately diverse; some plants are high-THCA or high-CBDA, and others are even-ratio chemovars (where the THCA:CBDA concentration is approximately 1:1).

Average cannabinoid concentrations are shown in Figure S.2.1 to highlight the trends of cannabinoid concentration over time. Minor cannabinoids of interest, such as CBGA, showed a peak in concentration in the first stage, but concentrations decreased rapidly in the second stage for most plants, likely due to CBGA being enzymatically converted into THCA and CBDA. CBN and CBNA share an oxidation pathway with THC and THCA, respectively, and subsequently, there was an increase in CBN concentrations over time. CBNA had a decrease in concentration over time, possibly due to light exposure resulting in CBNA degrading into CBN.

Categorizing the plant population by genotype (high-THCA, even-ratio, and high-CBDA) showed that concentrations of the minor cannabinoids varied based on genotype (Figures S2.2–2.4), as expected by cannabis biosynthesis pathways. Although CBGA peaked in the first amber stage for high-THCA and even-ratio genotypes, it peaked in the third amber stage for high-CBDA genotypes; possibly, there is a genotype-specific trait which converts CBGA to CBDA later for these plants. Although in low abundance, CBC concentrations peaked during Stage 2 for even-ratio (0.07 mg/g) and high-THCA (0.11 mg/g) genotypes, then declining in Stage 3, as opposed to the high-CBDA (0.03 mg/g) genotypes, which peaked in Stage 4. These findings are contradictory to previous studies in the literature, where CBC amounts did not decrease over time [29]; hence, some degradation of CBC must be occurring in even-ratio and high-THCA genotypes.

A previous study by Linder et al. [23] investigated two genotypes and found that over time, total CBD and THC concentrations increased. However, the authors did not investigate stigma color or how it correlates with CBD and THC concentration. The current study has investigated how stigma color is linked to cannabinoid production and demonstrated that, for most genotypes, Stage 3 (mostly amber) Stage 4 (fully amber) have the highest peak of TCC and TCY. The findings suggest that harvesting based upon stigma color has merit. Further investigation with a larger collection of genotypes would help to confirm how applicable our findings are across this very diverse species. Additionally, it would be beneficial for cultivators to carry out individual genotype studies on genotypes which may be of greater interest, as they look to optimize the harvest of cannabis inflorescences for maximum cannabinoid yield.

### 2.3. Cannabinoid Prediction Models

**Figure 8:**
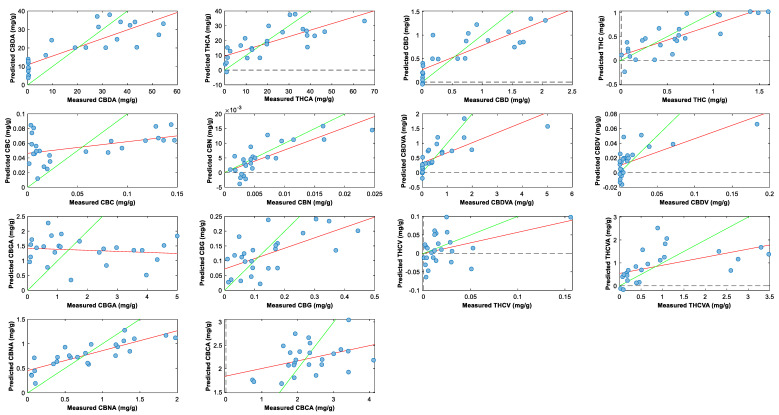
Plot of measured values versus predicted values for the determination of cannabinoid compounds in whole inflorescence (still attached to plant) using the PLS-R tool in PLS-Toolbox 9.0. Green line: line of best fit from calibration data; red line: line of best fit from predicted data. Preprocessing parameters: detrend; standard normal variate; mean centering.

The dataset consisted of 100 samples (4 inflorescences × 25 genotypes). This study investigated the effect of prediction models when using whole inflorescences before harvest against inflorescences after harvest without sugar leaves. For ease of reference, whole inflorescence before harvest is referred to as ‘whole inflorescence’, whilst inflorescence after harvest without sugar leaves is referred to as ‘trimmed inflorescence’. These prediction models used NIR spectra data and LCMS analysis data. Different preprocessing parameters were applied to optimize the prediction of 14 cannabinoid concentrations (Table 1, Figure 8). *R*^2^ values were calculated for the whole inflorescence data, where values closer to 1 indicate a strong prediction model. The *R*^2^ values are as follows: THC (0.70), CBDA (0.69), CBNA (0.69), CBD (0.67), CBN (0.63), THCA (0.55), and CBDVA (0.53). Predictions for TCC were built but performed poorly, with an *R*^2^ of 0.32. For trimmed inflorescences, *R*^2^ values improved for the main cannabinoids (Table 2), i.e., CBDA (0.84), THCA (0.69), CBD (0.74), and THC (0.75), and for some minor cannabinoids, i.e., CBG (0.57) and THCVA (0.56). Predictions for TCC were developed for both whole inflorescences (*R*^2^ = 0.32) before harvest and whole inflorescences without sugar leaves (*R*^2^ = 0.37) but performed poorly. Permutation tests were applied to all models (*n* = 200), where most models returned a *p*-value of less than 0.05 (Table S3 and Figure S3). Multiple preprocessing techniques were employed when developing all prediction models; different parameters and methodologies were applied with reference to the previous literature [24,30].

Residual prediction deviation (RPD) was determined to assess the model’s accuracy between observed values and predicted values. Values above 1.5 indicate a model has moderate predictive power, a value above 2.0 indicates a model has good predictive power, and a value above 2.5 indicates excellent predictive power. From the full inflorescence results, the RPD of five cannabinoids—CBDA (1.66), CBD (1.68), THC (1.72), CBN (1.50), and CBNA (1.56)—indicated moderate predictive power, whereas from the results for trimmed inflorescences, only four cannabinoids had RPD values over 1.5. However, it should be noted that the RPD values for CBDA (2.26), THCA (1.82), CBD (1.86), and THC (1.92) improved. Overall, these RPD values are quite good considering the heterogeneity of the sample. It is noteworthy that the THCA predictors and RPD (1.46) performed so poorly, because previous studies indicate that THCA should perform better due to its high abundance (20–80 mg/g). THCA usually has a higher *R*^2^ value than THC, its neutral form [30]. The authors postulate that this may be due to interference from other volatile compounds or moisture, which is affecting the NIR signals exclusive to THCA. A direct comparison between dried and non-dried inflorescences needs to be explored to confirm this. The poor performance of THCA relative to THC is consistent between whole and trimmed inflorescences.

Prediction models based on whole inflorescence before harvest for CBDA, THC, CBD, CBN, and CBNA provide sufficient predictive potential for cultivation settings. Being able to target all of the abundant and significant cannabinoids will greatly benefit cultivators as this will allow their cross-breeding selection process to become more efficient, being able to select cross-bred genotypes sooner for cultivation. Additionally, prediction models for CBDA and CBD mean that the work on cannabinoid prediction can be expanded to high-CBDA and hemp variety cannabis plants as well. This is of great interest to cultivators, as CBDA and CBD are medicinal targets, with CBDA often being targeted by cultivators for maximum production.

Similar research studies investigating the use of handheld portable NIR devices to build cannabinoid prediction models when analyzing whole cannabis inflorescences have been performed by Tran et al. [24,31] and Deddia et al. [25]; however, they were only able to produce prediction models for THCA or THC concentrations. This current study has expanded upon NIR cannabinoid prediction capabilities and models have thus been further developed for CBDA, CBD, CBN, CBNA, and CBDVA.

**Figure 9:**
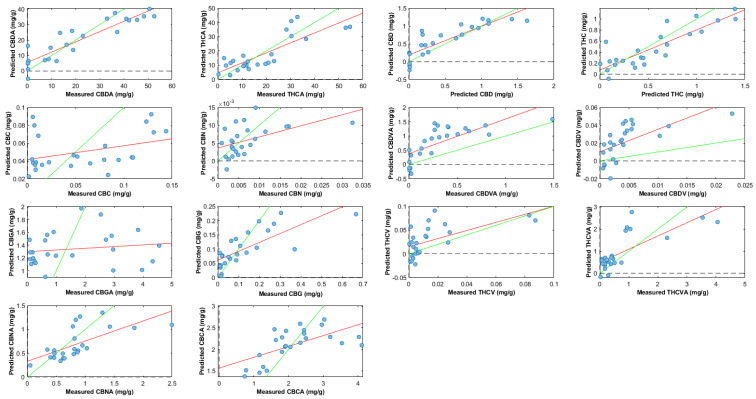
Plot of measured values versus predicted values for the determination of cannabinoid compounds in whole inflorescence without sugar leaves using the PLS-R tool. Green line: line of best fit from calibration data; red line: line of best fit from predicted data. Preprocessing parameters: Detrend; standard normal variate; mean centering.

Regression models were also built for inflorescences without sugar leaves (Table 2, Figure 9). Sugar leaves, which do not contain cannabinoids, are typically removed post-cultivation because they dilute the final cannabinoid concentration. A comparison was made between the whole inflorescence versus an inflorescence with sugar leaves removed to see which would produce better results, and it was found that the inflorescence with sugar leaves removed produced stronger prediction models. This is because the MicroNIR is exposed to more trichomes and therefore more cannabinoids for the NIR to scan. Sugar leaves tend to have a lot less trichomes on their surfaces and their inclusion within the scan may underestimate the results.

A model was developed to see whether the MicroNIR could predict the amber stage and amber score, but both models performed poorly. This is not surprising as the NIR is sensitive to organic compounds rather than colors on the visible light spectrum. It would have been ideal if the MicroNIR could be used to predict the optimal harvest time or amber score; however, NIR’s ability is limited to the endogenous concentrations of cannabinoids. Alternative assessment methods may be the use of hyperspectral imaging [32], which utilizes wavelengths within both the visible light spectrum and NIR spectrum. Hyperspectral imaging has the benefits of capturing the color change of stigmas whilst also capturing the NIR spectral data linked to endogenous concentrations of cannabinoids found on the surface of inflorescences (trichomes). Similar image analysis techniques need to be applied to distinguish the stigmas from the trichomes.

Finally, a model was developed to predict TCY but also yielded poor results. The MicroNIR was able to predict the concentrations of CBDA, THCA, CBD, THC, CBN, and CBNA, which account for 90% of the TCC. By utilizing prior information from plant studies, cultivators should be able to make informed decisions on whether a plant has peaked in concentration. Additionally, totaling the predicted values from CBDA, THCA, CBD, THC, CBN, and CBNA models, we can obtain an approximation of 90% of the total cannabinoid content.

## 3. Materials and Methods

### 3.1. Cultivation and Sample Preparation

All plants were cloned from seed-grown plants obtained from Agriculture Victoria Research. All the work undertaken was performed under a Medicinal Cannabis Research License (MC029) and Permits (MC029-S01-C03) issued by the Department of Health and Aged Care, Office of Drug Control (ODC), Australia.

The experiment consisted of 25 diverse genotypes with a mixture of chemovars: 9 high-THCA, 5 high-CBDA, and 11 even-ratio plants. High-THCA genotypes contained at least 3 times the concentration of THCA to CBDA. High-CBDA genotypes contained at least 3 times the concentration of CBDA to THCA. Even-ratio genotypes contained generally even concentrations of CBDA and THCA, but no more than 3 times the concentration of each, i.e., high THCA < 1:3; high CBDA > 3:1; 3:1 > even ratio > 1:3 (x:x = CBDA:THCA ratio).

**Figure 10:**
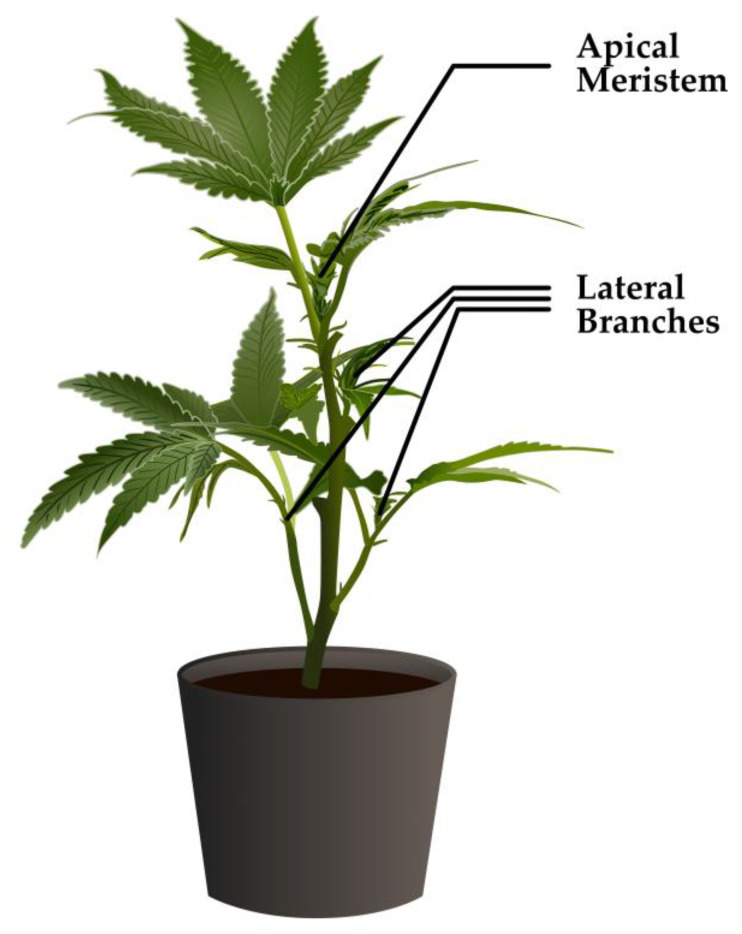
Schematic diagram of a typical young cannabis seedling showing the position of the apical meristem and lateral branches. In this experiment, the apical meristem was removed and four lateral branches retained.

Cannabis plants were struck from cuttings which were 10 cm in length and were cut diagonally with 3 nodes for each cutting. The lower cut was dipped into Clonex (Growth Technology, Taunton, UK) rooting hormone gel and potted into growing trays containing 5 cm coir pellets (Jiffy Products S.L. Ltd., Mirigama, Sri Lanka). Cuttings typically rooted within a week and would be left to grow for a total of 4 weeks in a controlled-temperature environment at 25 °C at 60% relative humidity. The plants were transferred into 20 cm diameter pots filled with potting mixture containing vermiculite, perlite, water-holding granules, and garden lime. The plants were grown in three Pinelab 4 × 8 grow tents (Skah International Pty Ltd., Adelaide, Australia) equipped with an Inkbird ITC-308 temperature controller (Inkbird, Shenzhen, China) set to 25 °C; the humidity was monitored to be around 60%. Each tent contained 8–9 plants. Lighting was provided by a 600 W high-pressure sodium lamp and a 1000 W ceramic metal-halide lamp housed in a HI-PAR Duo Reflector (HI-PAR, Adelaide, Australia). Plants were given Controlled Release Fertilizer Macracote Coloniser Plus (Langely Fertilizers, Perth, Australia) 12:4:10 (N:P:K) and watered with liquid-soluble fertilizer 4:2:7 (N:P:K) containing a mixture of Total Horticultural Concentrate Part A Part B (THC Australia, Victoria, Australia) diluted to maintain an electrical conductivity (EC) of 1.0 and pH of 6.5. During the growth/vegetation stage, the plants were grown under a 16:8 day–night cycle. The apical meristem was removed, and the plant was pruned down to four lateral branches (Figure 10). Any secondary lateral shoots that formed along the four branches were also removed to ensure that energy was directed into the cola (floral) regions. The plants were grown for a total of 4 months before floral initiation was commenced.

Once plants reached the flowering stage, they were moved to a 12h : 12h day–night cycle. The inflorescences on each plant were harvested based on the color progression of the stigmas. This color change, from white to amber, is categorized into four stages determining their harvest order. A visual (manual) assessment of each inflorescence was performed, with the aim of classifying it as belonging to one of four stages based on the color of its stigmas. The classifications are subjective and based on an overall perception of the color of the stigmas across an individual inflorescence, as well as an assessment of the number of stigmas that have started to turn amber, given that an inflorescence will mature in stages. Inflorescences classified as being in stage 1 had only white stigmas; those in stage 2 had an off-white or light yellowing color on some of the stigmas; stage 3 had a deeper yellow color and a majority of stigmas that had started to yellow; stage 4 had a deep amber color on all of the stigmas. Sample harvesting based on amber score occurred across 119 days after flowering initiation. All harvest dates, as well as the mean and median for each stage, have been recorded and put in Supplementary Tables S4 and S5 for reference.

### 3.2. Image Analysis and Color Quantification

Digital RGB images of the inflorescences were taken from an axial view using a 1300D Canon DLSR Camera (Canon Inc., Ota, Tokyo, Japan), which was mounted on a Kaiser RS1 Copy Stand (Kaiser Fototechnik, Buchen, Germany). Two Kaiser RB 5070 DX2 Copylizer LED lights (Kaiser Fototechnik, Buchen, Germany) provided the only source of illumination. The lights were positioned either side of the sample and pointed at an angle parallel to the table. The camera was set to have a focal length of 35 mm, aperture f/16, exposure 1/30 s, and ISO 100. RGB images were saved in JPEG format with dimensions of 5184 × 3456 pixels. The camera was positioned at a height of 30 cm from the base of the copy stand.

**Figure 11:**
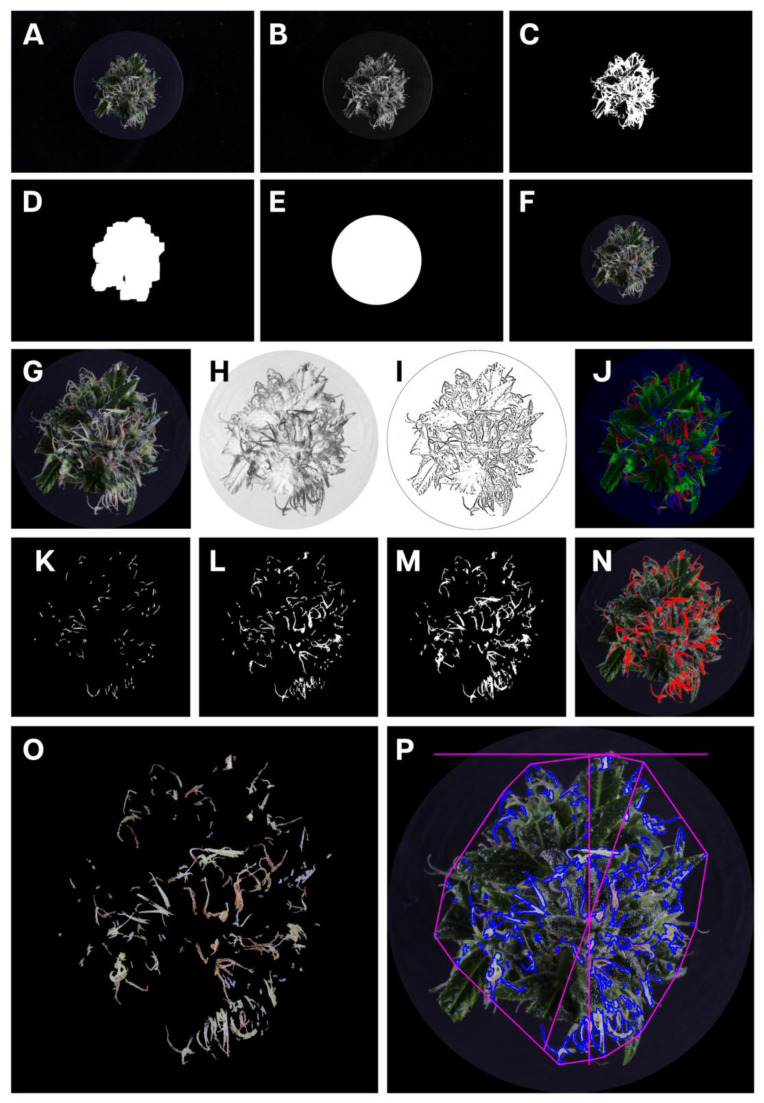
Representation of stages in the image analysis pipeline used to isolate stigmas from the cannabis inflorescence for digital analysis. (**A**) The original RGB image showing the inflorescence; (**B**) the RGB image is converted to the L*a*b color space; (**C**) the L-channel was isolated and a binary threshold of 41 applied to separate the inflorescence from the background; (**D**) a 27 × 27 opening operation was applied, followed by a 41 × 41 dilation operation to create a binary mask; (**E**) a minimum enclosing circle was placed around the binary mask to create a circular mask; (**F**) the circular mask was applied to the RGB image to remove unnecessary detail outside of the inflorescence; (**G**) a minimum enclosing rectangle is fitted around the binary mask to crop the image to improve analysis efficiency; (**H**) the modified RGB image was converted into the CMYK color and a median blur of 11 px applied to the K-channel; (**I**) an adaptive threshold was applied to the K-channel, using a Gaussian-weighted sum of the neighborhood values with a neighborhood area of 11; (**J**) to assist with trichome removal and small stigma retention, a maxRGB operation was applied to the RGB image; (**K**) the B-channel from the maxRGB image was isolated to identify trichomes; (**L**) a logical-AND operation combined the maxRGB binary with the binary image that had been subjected to the adaptive thresholding to collect all of the stigmas; (**M**) a logical-OR operation was then used to combine this with the previously derived binary image to create a final binary mask; (**N**) an illustration showing how the stigmas have been correctly identified, indicated as red markings overlaying the original cropped RGB image; (**O**) the final binary mask is applied to the cropped RBG image to retain only the stigmas; (**P**) the image can then be analyzed for various morphological and color characteristics.

A customized PlantCV [33,34] image analysis pipeline was written to perform the image analysis steps. PlantCV version 4.4.0 running with OpenCV version 4.10.0.84 and Python version 3.12.6 under the Linux Ubuntu 22.04.5 operating system was used to perform the analysis. RGB images (Figure 11A) were loaded into the pipeline and converted into the L*a*b color space (Figure 11B). The L-channel was isolated and a binary threshold of 41 applied to separate the inflorescence from the background (Figure 11C). As the lighting was held constant across all images, this was easily accomplished by thresholding images to a constant gray level. A 27 × 27 opening operation was applied, followed by a 41 × 41 dilation operation to create a final binary mask (Figure 11D), which was then applied to the RGB image by fitting a minimum enclosing circle around the mask (Figure 11E) and replacing all unnecessary detail from the image with black pixels (Figure 11F). Following this, a minimum enclosing rectangle was fitted around the binary blob to clip the RGB image and speed up processing (Figure 11G).

Next, the modified RGB image was converted into the CMYK (cyan, magenta, yellow, black) color space (Figure 11H) and a median blur of 11 px applied to the K-channel. Next, an adaptive threshold was applied, using a Gaussian-weighted sum of the neighborhood values with a neighborhood area of 11 (Figure 11J). To assist with trichome removal and small stigma retention, a maxRGB operation [35] was applied to the RGB image (Figure 11K) and the B channel was isolated. The B channel was subjected to a binary threshold of 111 (Figure 11L). A logical-AND operation combined this with the binary image that had been subjected to the adaptive thresholding (Figure 11M). A 5 × 5 morphological closing operation was then applied before the resultant binary image was combined with a copy of the maxRGB binary image via a logical-XOR operation. In order to return large stigmas, it was necessary to convert a copy of the RGB image into the CMYK color space, apply a binary threshold of 110 to the K-channel, and then invert the image. A logical-OR operation was then used to combine this with the previously derived binary image to create a final binary mask (Figure 11N) of the stigmas, which was applied to the original RGB image (Figure 11N–P).

To digitally quantify the color of the stigmas, a pixel-wise analysis was performed using the L*a*b color model based on a modification of the ‘greenness’ index described in Depetris et al. [36]. For the current work, a ‘yellowness’ index was created instead, using the same methodology, but with the *L* and *b* channels to create a normalized index ranging from white (0) to yellow (100). This formed the automated amber score.

### 3.3. LCMS Analysis

Harvested inflorescences were placed into grinding jars, which were immediately frozen using liquid nitrogen and then ground to a fine powder using the SPEX SamplePrep 2010 Geno/Grinder (SPEX SamplePrep, Metuchen, NJ, USA) for 1 min at 1200 rpm. Afterwards, all samples were freeze-dried for 48 h using a VirTis General Purpose Freeze Dryer (Scientific Products, Warminster, PA, USA). A subsample of 10 mg was taken for LCMS analysis, as outlined in Elkins et al. (2019) [37]. Analysis was performed on a Thermo Scientific (Waltham, MA, USA) Q Exactive Plus Orbitrap mass spectrometer (MS) coupled with a Thermo Scientific Vanquish ultra-high-performance liquid-chromatography (UHPLC) system equipped with a degasser, binary pump, temperature-controlled autosampler and column compartment, and diode array detector (DAD). CBD, THC, CBC, CBN, CBDVA, CBDV, CBGA, CBG, THCV, THCVA, CBNA, and CBCA were quantified using the MS, except for CBDA and THCA, which were quantified using the UV-DAD. TCC was the sum of all 14 cannabinoid concentration results.

### 3.4. NIR Spectroscopy

A VIAVI MicroNIR Onsite-W (VIAVI Solutions Inc., Scottsdale, AZ, USA) was utilized for scanning and data were collected within the 950–1650 nm range, with a resolution of 6.2 nm. The handheld portable MicroNIR uses two integrated vacuum tungsten lamps with a 128-pixel InGaAs photodiode array detector. The dispersing element is a VIAVI linear variable filter (LVF) (VIAVI Solutions Inc., Scottsdale, AZ, USA) and the windowed measurement collar uses an anti-reflective sapphire window. The signal-to-noise ratio is 25,000 and the integration time is 10 ms. The portability and field deploy-ability allows it to be used in the field or within growing facilities. The device was configured in diffuse reflectance mode with an integration time of 12 ms and 100 scan counts. Data acquisition was performed using MicroNIR Pro v3.0 software (VIAVI Solutions Inc., Scottsdale, AZ, USA). In total, 100 cannabis samples were scanned in triplicate (25 genotypes × 4 inflorescences per genotype). The detector was thoroughly cleaned using 80% methanol (Thermo Fisher Scientific Inc., Waltham, MA, USA) (methanol/water) and a KimTech Kim-Wipe tissue (Kimberly-Clark, Irving, TX, USA) between all scans until all residue was removed. Scans were performed on cannabis inflorescences before harvest in triplicate, scanning a different section of the inflorescence each time, and the resulting data were averaged. The inflorescences were harvested, had sugar leaves removed, and were re-scanned again in triplicate.

### 3.5. Statistical Analysis

The LCMS data were processed, and graphs were produced using Microsoft Excel (Microsoft Corporation, Redmond, WA, USA). Data visualizations were prepared using RStudio v2024.04.2 + 764 (Posit PBC, Boston, MA, USA) with the ggplot2 package [38].

### 3.6. Cannabinoid Prediction Models Using NIR

VIAVI MicroNIR Onsite-W spectra data were exported from MicroNIR Pro v3.0 and imported into Microsoft Excel, where the mean data from the three scans were calculated. Data were then imported into MATLAB 2022a (Mathworks, Natick, MA, USA) with PLS-Toolbox 9.0 (Eigenvector Research Inc, Manson, WA, USA) for analysis. For statistical modeling, preprocessing used a combination of detrend, second-order derivate, standard normal variate (SNV), and mean centering to obtain the strongest model based on *R*^2^ prediction value (Figure 2). Partial least squares regression models used LCMS quantitative data as dependent variables and NIR data from the VIAVI MicroNIR Onsite-W as independent variables. Venetian blind cross-validation was used within 10 splits and blind thickness set to 1.

The dataset was separated into calibration and validation sets where 75% (*n* = 75) of the data was used to train the model and 25% (*n* = 25) of the data was used to test the model. The Kennard–Stone algorithm was used to ensure the data selected for the calibration set were uniform and representative of the whole dataset, while the validation set contained samples that are interior and exterior to the calibration set. This eliminates extrapolation of the calibration model when applied to the validation test. The same calibration and validation dataset split was used for all models. To test for statistical significance, permutation tests (*n* = 200) were performed for all models (Wilcoxon, Sign Test, and Rand *t*-test).

## 4. Conclusions

This study has provided a useful insight into the relationship between maximum cannabinoid production and optimal harvest time based on stigma color transition across multiple unique genotypes. The cannabis industry currently chooses to harvest plant based upon stigma color, when most stigmas are amber. Given our findings, this is certainly applicable to most genotypes. We found that higher amber scores correlate with higher yields for the majority of the genotypes that we tested. Additionally, prediction models for THCA, THC, CBDA, CBD, CBN, CBNA, and CBDVA concentrations have been developed for early screening of whole cannabis inflorescences, which will greatly benefit growers in making rapid decisions for cross-breeding programs and large-scale production.

We recommend that a longer-term study of cannabinoid biosynthesis patterns and stigma color for each individual genotype be undertaken to determine the optimal harvest time for each. If such a study were conducted, the isolation of the genetic traits which cause peaks in cannabinoid concentration in early flowering stages would be of great value to the industry, improving efficiency and overall productivity.

## Figures and Tables

**Table 1:** Partial least squares regression models (PLS-R) for the determination of cannabinoid compounds in whole inflorescence still attached to the plant using data from the NIR (*n* = 100).

	RMSEC ^1^	*R*^2^_Cal_ ^2^	RMSECV ^3^	*R* ^2^_CV_^4^	RMSEP ^5^	Pred Bias ^6^	*R* ^2^_Pred_^7^	RPD ^8^
CBDA	11.91	0.61	13.53	0.50	11.65	0.55	0.69	1.66
THCA	8.11	0.73	9.63	0.63	12.05	−2.44	0.55	1.46
CBD	0.45	0.64	0.52	0.53	0.42	−0.04	0.67	1.68
THC	0.41	0.64	0.53	0.40	0.28	−0.10	0.70	1.72
CBC	0.04	0.25	0.05	0.11	0.05	0.00	0.20	1.13
CBN	0.01	0.54	0.01	0.24	0.00	0.00	0.63	1.50
CBDVA	0.61	0.55	0.70	0.41	0.78	−0.09	0.53	1.39
CBDV	0.04	0.40	0.04	0.16	0.03	0.00	0.46	1.36
CBGA	1.14	0.20	1.24	0.07	1.74	−0.50	0.02	0.91
CBG	0.09	0.51	0.10	0.33	0.09	−0.02	0.39	1.27
THCV	0.09	0.35	0.12	0.02	0.04	−0.01	0.18	0.78
THCVA	0.89	0.51	1.08	0.30	0.91	−0.06	0.27	1.18
CBNA	0.30	0.59	0.36	0.43	0.36	−0.02	0.69	1.56
CBCA	0.66	0.43	0.73	0.32	0.73	−0.08	0.16	1.10
TCC	14.35	0.54	16.12	0.42	13.17	1.67	0.32	1.19

CBDA: cannabidiolic acid; THCA: tetrahydrocannabinolic acid; CBD: cannabidiol; THC: tetrahydrocannabinol; CBC: cannabichromene; CBN: cannabinol; CBDVA: cannabidivaric acid; CBDV: cannabidivarin; CBGA: cannabigerolic acid; CBG: cannabigerol; THCV: tetrahydrocannabidivarin; THCVA: tetrahydrocannabidivarinic acid; CBNA: cannabinolic acid; CBCA: cannabichromenic acid; TCC: total cannabinoid concentration. The scan region was in the 950–1650 nm range. The preprocessing parameters used were detrend, standard normal variate, and mean centering. The derivative pre-treatment used was 2, 2, 5, where the first digit is the polynomial order, the second digit is the derivative order, and the third digit is the data point gap for which the derivative is calculated. The latent variable (LV) was 6. The number of unique samples (*n*) was 100 (the dataset was split into a calibration (*n* = 75) and validation set (*n* = 25) using the Kennard–Stone algorithm and used for all models). ^1^ RMSEC: root mean standard error of calibration. ^2^ *R*^2^_Cal_: coefficient of determination of calibration. ^3^ RMSECV: root mean standard error of cross-validation. ^4^ *R*^2^_CV_: coefficient of determination of cross validation. ^5^ RMSEP: root mean standard error of prediction. ^6^ Pred Bias: calculated prediction bias. ^7^ *R*^2^_Pred_: coefficient of regression of measured data vs. predicted data. Permutation testing (*n* = 200) using Wilcoxon, Sign test, and Rand *t*-test returned a *p*-value < 0.05 for cross-validation results. ^8^ RPD: residual prediction deviation.

**Table 2:** The partial least squares regression models (PLS-R) for the determination of cannabinoid compounds in whole inflorescence without sugar leaves using data from the NIR.

	RMSEC ^1^	*R*^2^_Cal_ ^2^	RMSECV ^3^	*R*^2^_CV_ ^4^	RMSEP ^5^	Pred Bias ^6^	*R* ^2^_Pred_^7^	RPD ^8^
CBDA	9.87	0.74	12.31	0.60	8.25	−1.43	0.84	2.26
THCA	8.22	0.76	10.00	0.64	8.06	−0.96	0.69	1.82
CBD	0.38	0.78	0.49	0.63	0.26	0.09	0.74	1.86
THC	0.46	0.56	0.55	0.38	0.22	−0.07	0.75	1.92
CBC	0.04	0.29	0.05	0.15	0.04	−0.01	0.14	1.09
CBN	0.01	0.33	0.01	0.13	0.01	0.00	0.25	1.15
CBDVA	0.68	0.58	0.85	0.36	0.60	0.45	0.52	0.57
CBDV	0.04	0.41	0.05	0.12	0.02	0.02	0.39	0.20
CBGA	1.25	0.11	1.44	0.00	1.49	−0.26	0.02	1.02
CBG	0.08	0.49	0.09	0.35	0.11	−0.03	0.57	1.34
THCV	0.10	0.22	0.13	0.01	0.03	0.01	0.36	0.79
THCVA	0.80	0.61	0.99	0.41	0.71	0.19	0.56	1.47
CBNA	0.26	0.72	0.31	0.60	0.40	−0.15	0.44	1.27
CBCA	0.70	0.31	0.82	0.11	0.74	−0.06	0.38	1.25
TCC	14.54	0.50	15.95	0.40	14.54	0.22	0.37	1.27

CBDA: cannabidiolic acid; THCA: tetrahydrocannabinolic acid; CBD: cannabidiol; THC: tetrahydrocannabinol; CBC: cannabichromene; CBN: cannabinol; CBDVA: cannabidivaric acid; CBDV: cannabidivarin; CBGA: cannabigerolic acid CBG: cannabigerol; THCV: tetrahydrocannabidivarin; THCVA: tetrahydrocannabidivarinic acid; CBNA: cannabinolic acid; CBCA: cannabichromenic acid; TCC: total cannabinoid concentration. The scan region was in the 950–1650 nm range. The preprocessing parameters used were detrend, standard normal variate, and mean centering. The derivative pre-treatment used was 2, 2, 5, where the first digit is the polynomial order, the second digit is the derivative order, and the third digit is the data point gap for which the derivative is calculated. The latent variable (LV) was 8. The number of unique samples (*n*) was 100 (the dataset was split into a calibration (*n* = 75) and validation set (*n* = 25) using the Kennard–Stone algorithm and used for all models. ^1^ RMSEC: root mean standard error of calibration. ^2^ *R*^2^_Cal_: coefficient of determination of calibration. ^3^ RMSECV: root mean standard error of cross-validation. ^4^ *R*^2^_CV_: coefficient of determination of cross validation. ^5^ RMSEP: root mean standard error of prediction. ^6^ Pred Bias: calculated prediction bias. ^7^ *R*^2^_Pred_: coefficient of regression of measured data vs. predicted data. Permutation testing (*n* = 200) using Wilcoxon, Sign test, and Rand *t*-test returned a *p*-value < 0.05 for cross-validation results. ^8^ RPD: residual prediction deviation.

## Data Availability

Raw data are available upon request.

## Supplementary Materials

The following supporting information can be downloaded at https://www.mdpi.com/article/10.3390/plants14101532/s1, Table S1: Liquid chromatography–mass spectrometry data for all cannabinoids analyzed in the harvest optimization trial (*n* = 100). Table S2: The inflorescence weight and cannabinoid yield of all sample inflorescences in the harvest optimization trial (*n* = 100). Figures S1.1–S1.14: Cannabinoid compound concentrations over four growth stages in 25 unique genotypes. Figures S2.1–2.4: Average cannabinoid concentration of each cannabinoid in each plant over four amber stages split between genotype type. Table S3 and Figure S3: Permutation results of PLSR models for full and trim inflorescences. Table S4: Harvest times by day for each inflorescence per genotype. Table S5: The mean and median harvest days for each amber stage.

## Abbreviations

The following abbreviations are used in this manuscript:

CBC	Cannabichromene
CBCA	Cannabichromenic acid
CBD	Cannabidiol
CBDA	Cannabidiolic acid
CBDV	Cannabidivarin
CBDVA	Cannabidivaric acid
CBG	Cannabigerol
CBGA	Cannabigerolic acid
CBN	Cannabinol
CBNA	Cannabinolic acid
CIELAB	LAB color space defined by the International Commission on Illumination
EC	Electrical conductivity
LCMS	Liquid chromatography–mass spectrometry
LV	Latent variable
NIR	Near-infrared
PLS	Partial least squares
*R*^2^cal	Coefficient of determination of calibration
*R*^2^cv	Coefficient of determination of cross-validation
*R*^2^pred	Coefficient of determination of prediction
RMSEC	Root mean squared error of calibration
RMSECV	Root mean squared error of cross-validation
RMSEP	Root mean squared error of prediction
RPD	Residual prediction deviation
TCC	Total cannabinoid concentration
TCY	Total cannabinoid yield
THC	Tetrahydrocannabinol
THCA	Tetrahydrocannabinolic acid
THCV	Tetrahydrocannabidivarin
THCVA	Tetrahydrocannabidivarinic acid
UV-DAD	Ultra-Violet Diode Array Detector
