# Supplementary material for: Determination of Optimal Harvest Time in Cannabis sativa L. Based upon Stigma Color Transition
Source: Plants (Basel). 2025 May 20;14(10):1532. doi: 10.3390/plants14101532 (PMC12114869; doi:10.3390/plants14101532)
Supplement: Supplementary file 1 [file plants-14-01532-s001.zip › Figure S1 Individual Cannabinoid Concentrations of all plants.pdf]

Figure S1.1 Cannabidiol (CBD) concentrations over four growth stages in 25 unique cultivars.

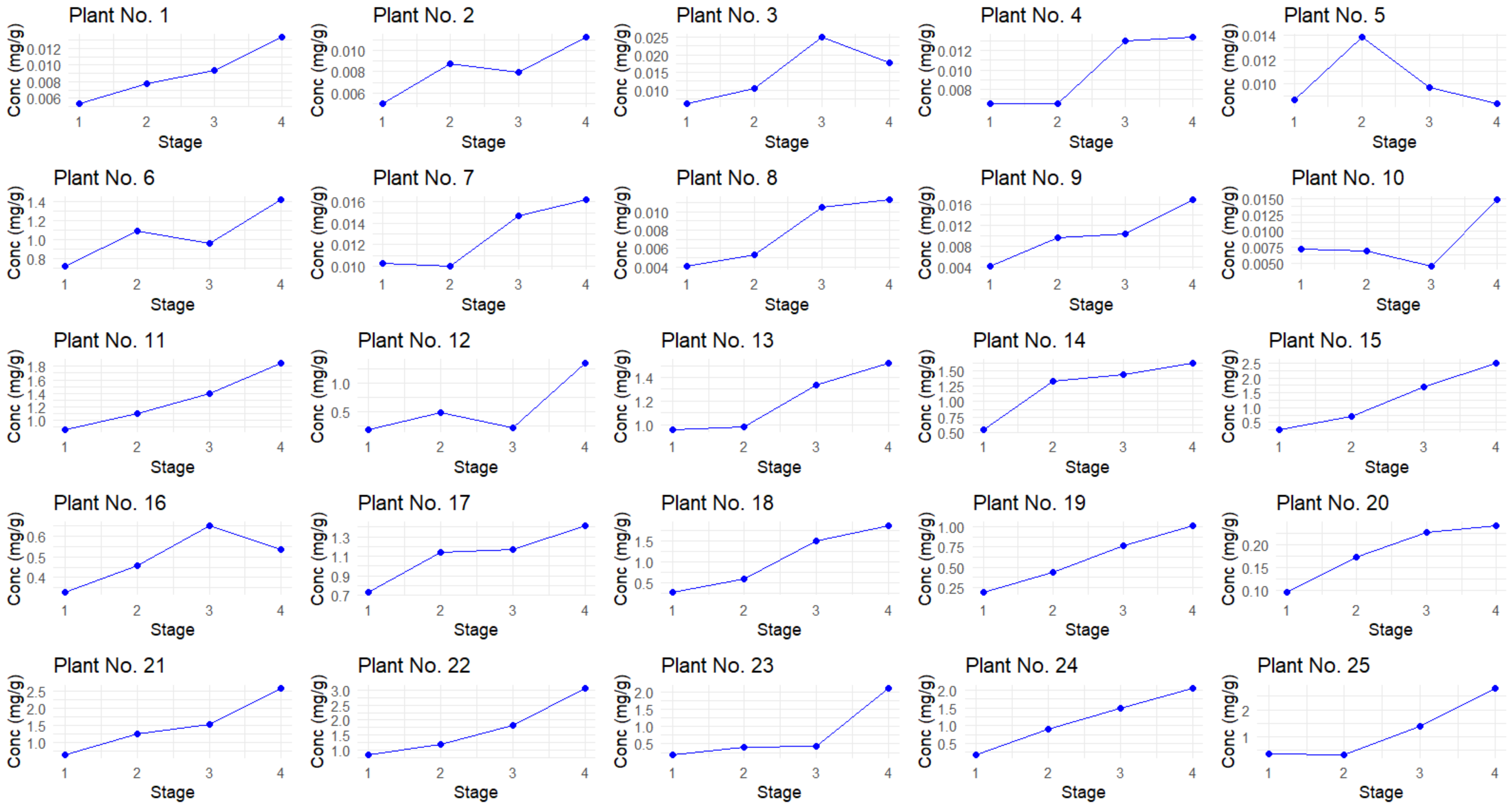

Unofficial

Figure S1.2 Cannabidiolic Acid (CBDA) concentrations over four growth stages in 25 unique cultivars.

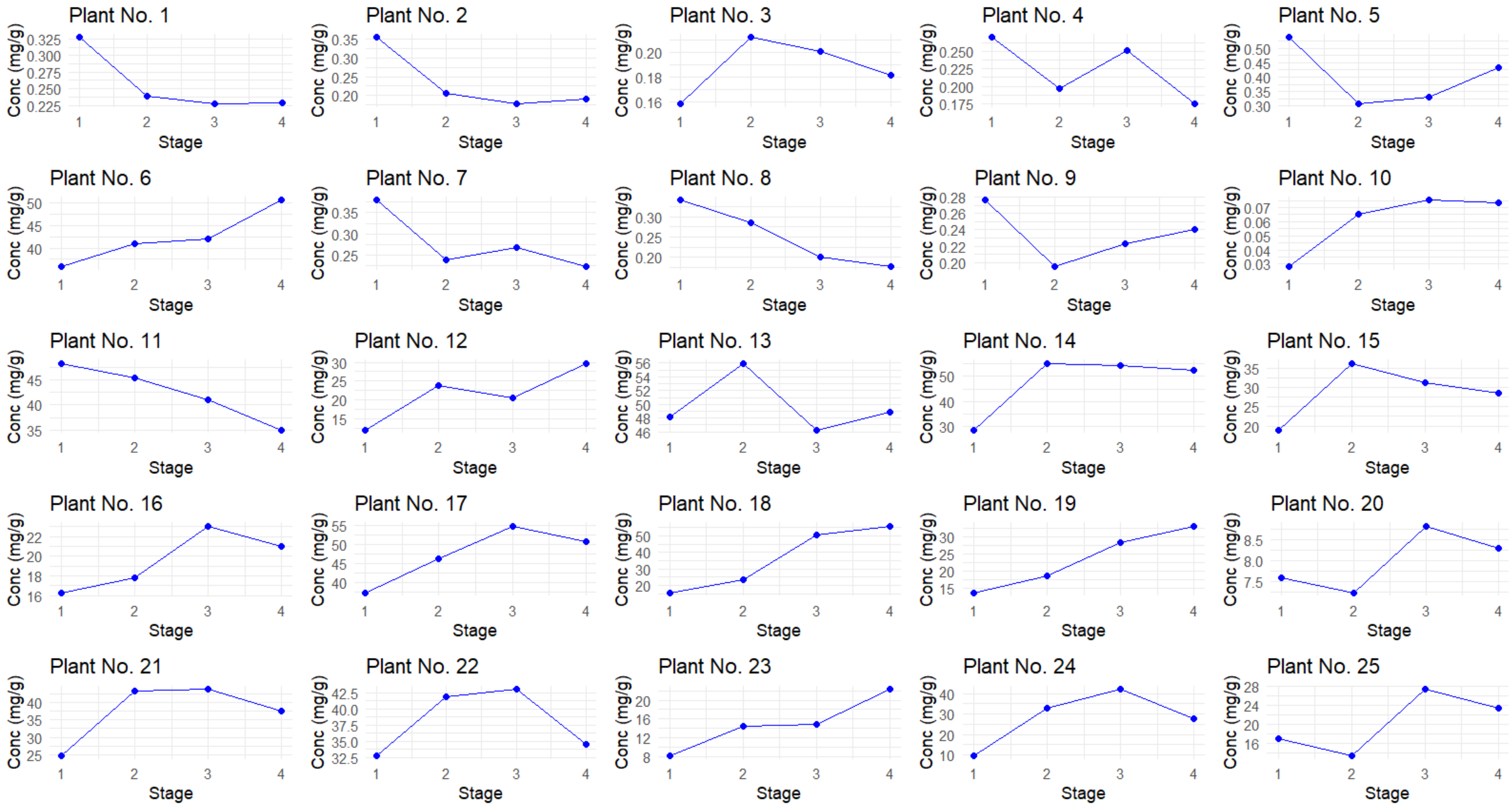

Unofficial

Figure S1.3 Cannabidivarin (CBDV) concentrations over four growth stages in 25 unique cultivars.

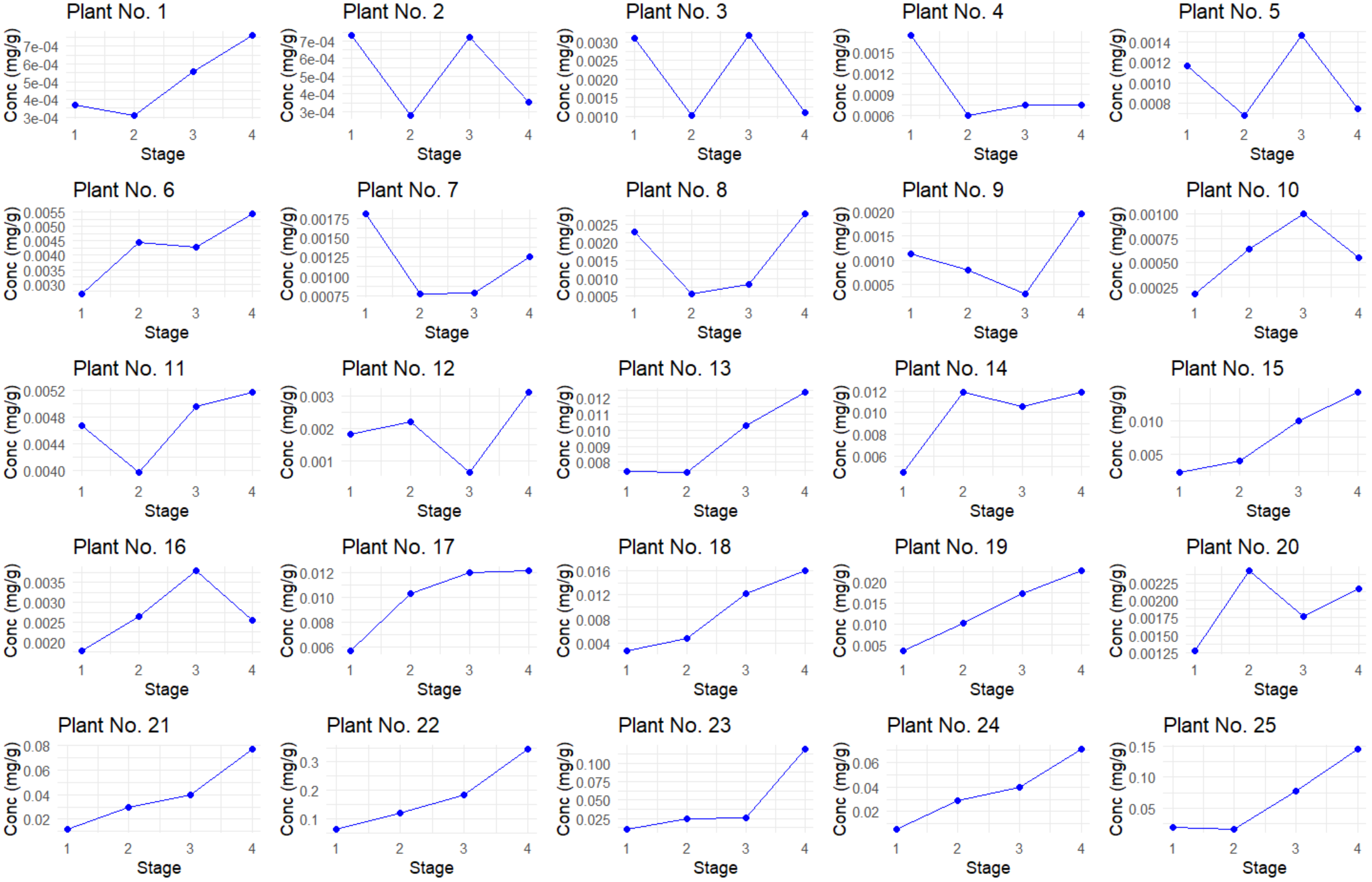

Unofficial

Figure S1.4 Cannabidivarinic Acid (CBDVA) concentrations over four growth stages in 25 unique cultivars.

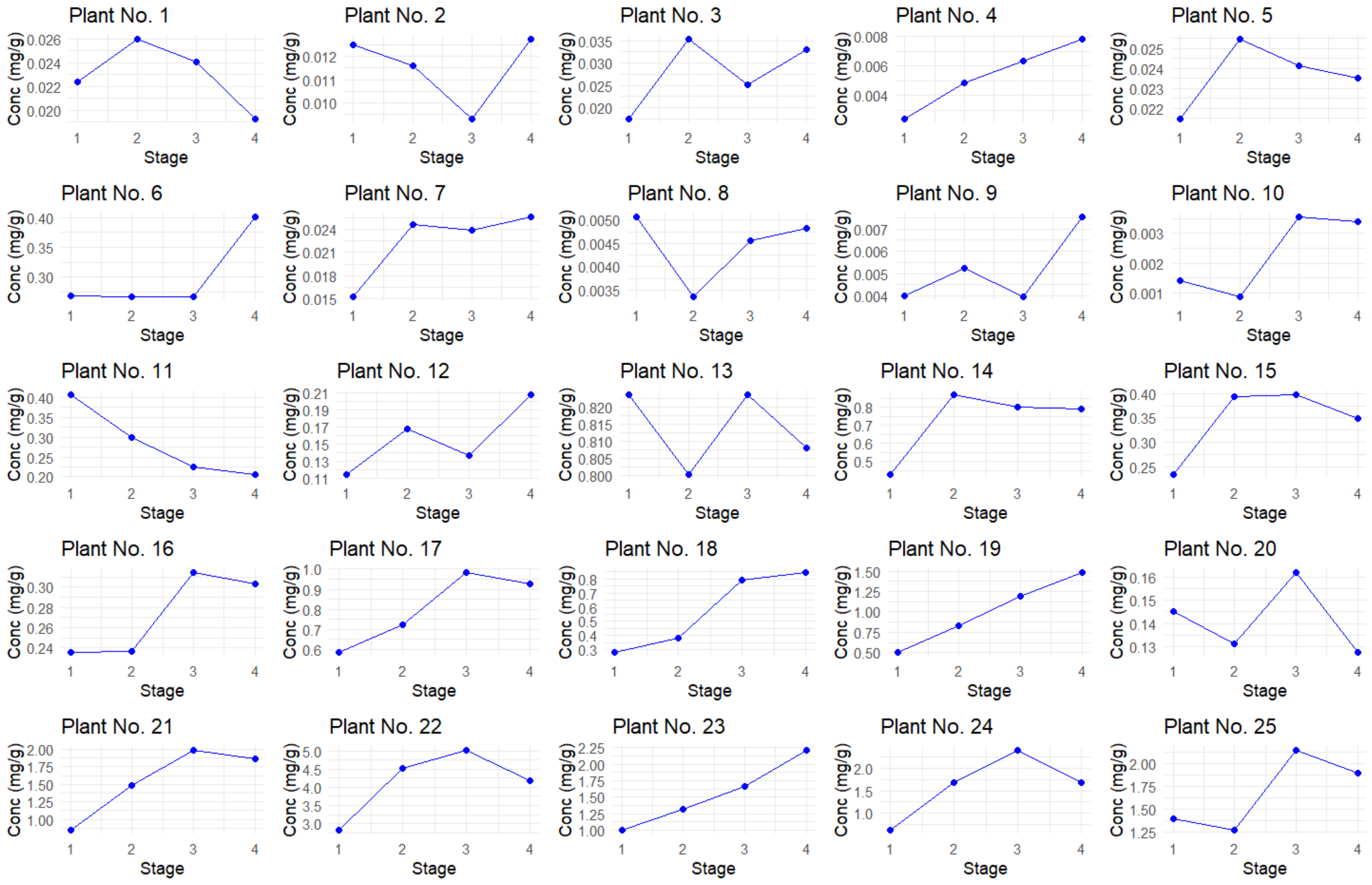

Unofficial

Figure S1.5 Cannabigerol (CBG) concentrations over four growth stages in 25 unique cultivars.

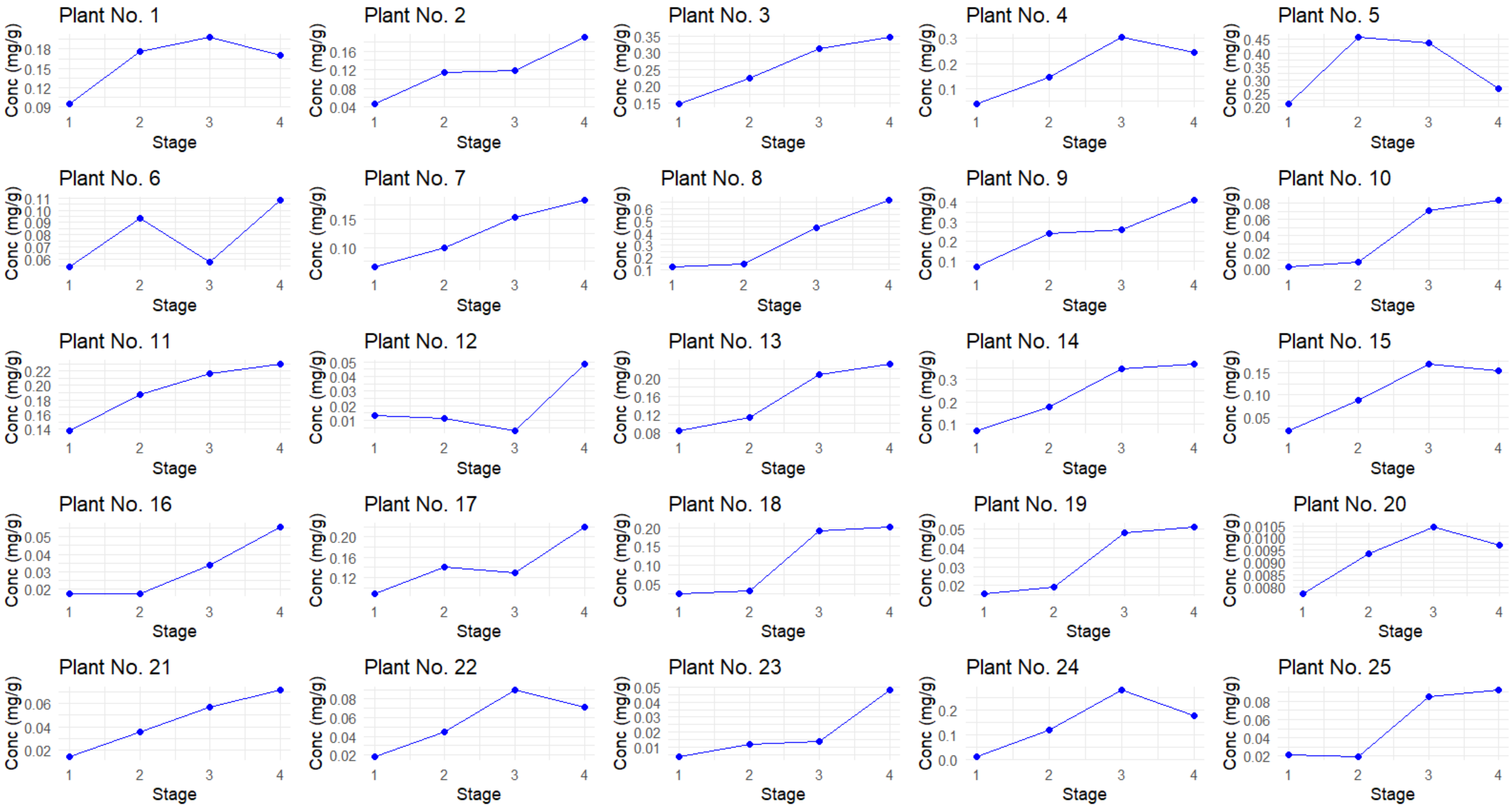

Unofficial

Figure S1.6 Cannabigerolic Acid (CBGA) concentrations over four growth stages in 25 unique cultivars.

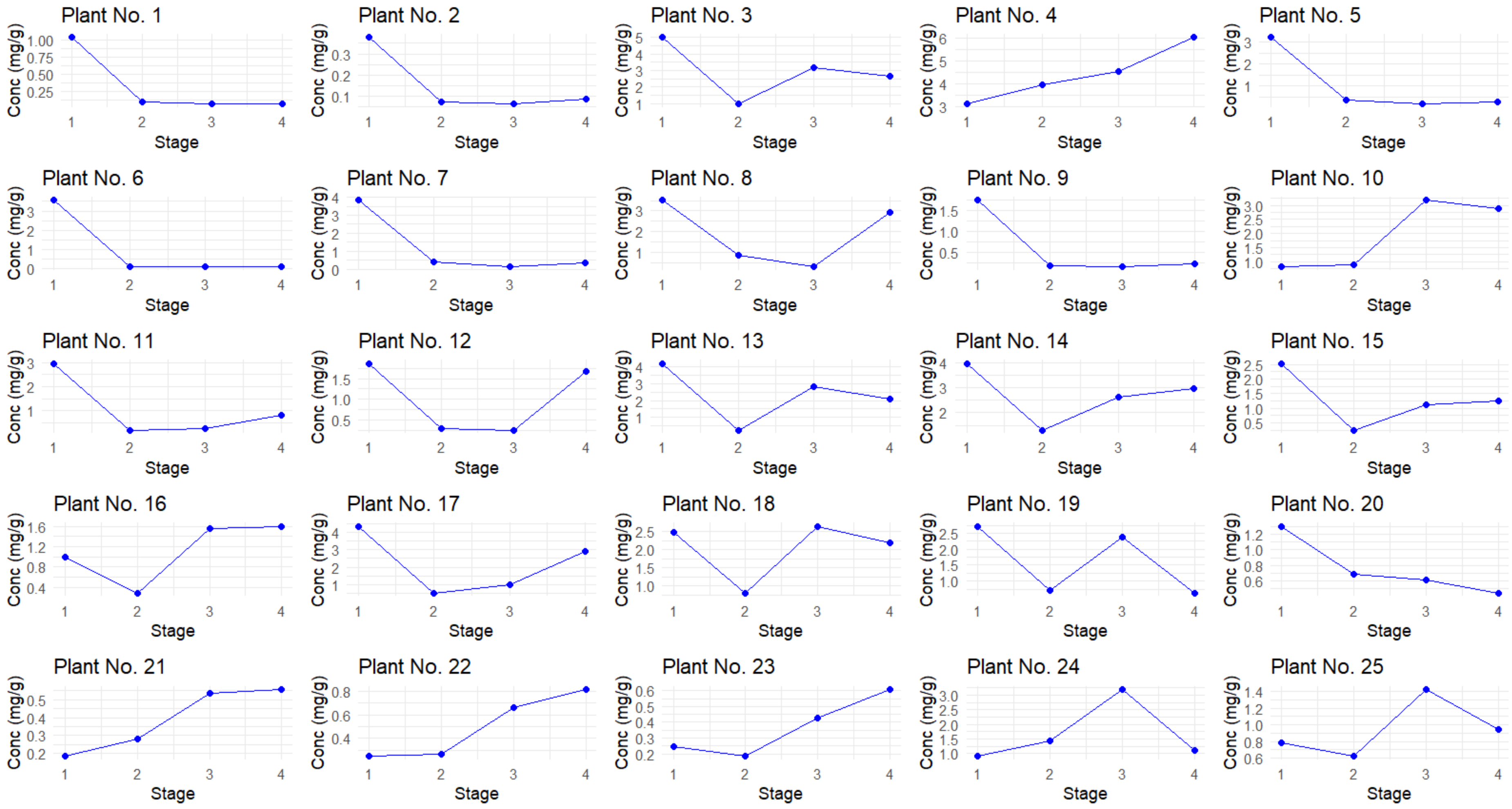

Unofficial

Figure S1.7 Cannabinol (CBN) concentrations over four growth stages in 25 unique cultivars.

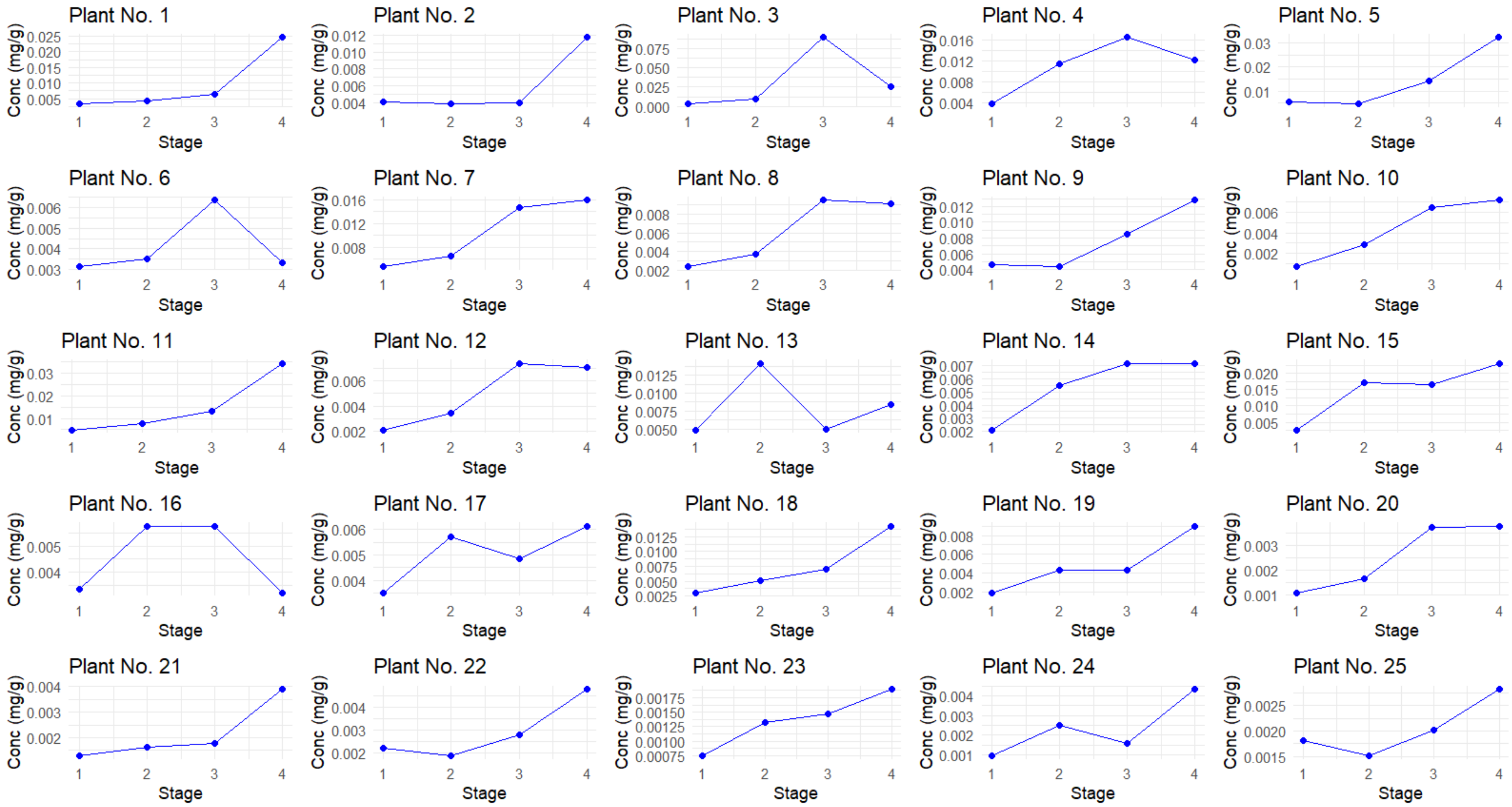

Unofficial

Figure S1.8 Cannabinolic Acid (CBNA) concentrations over four growth stages in 25 unique cultivars.

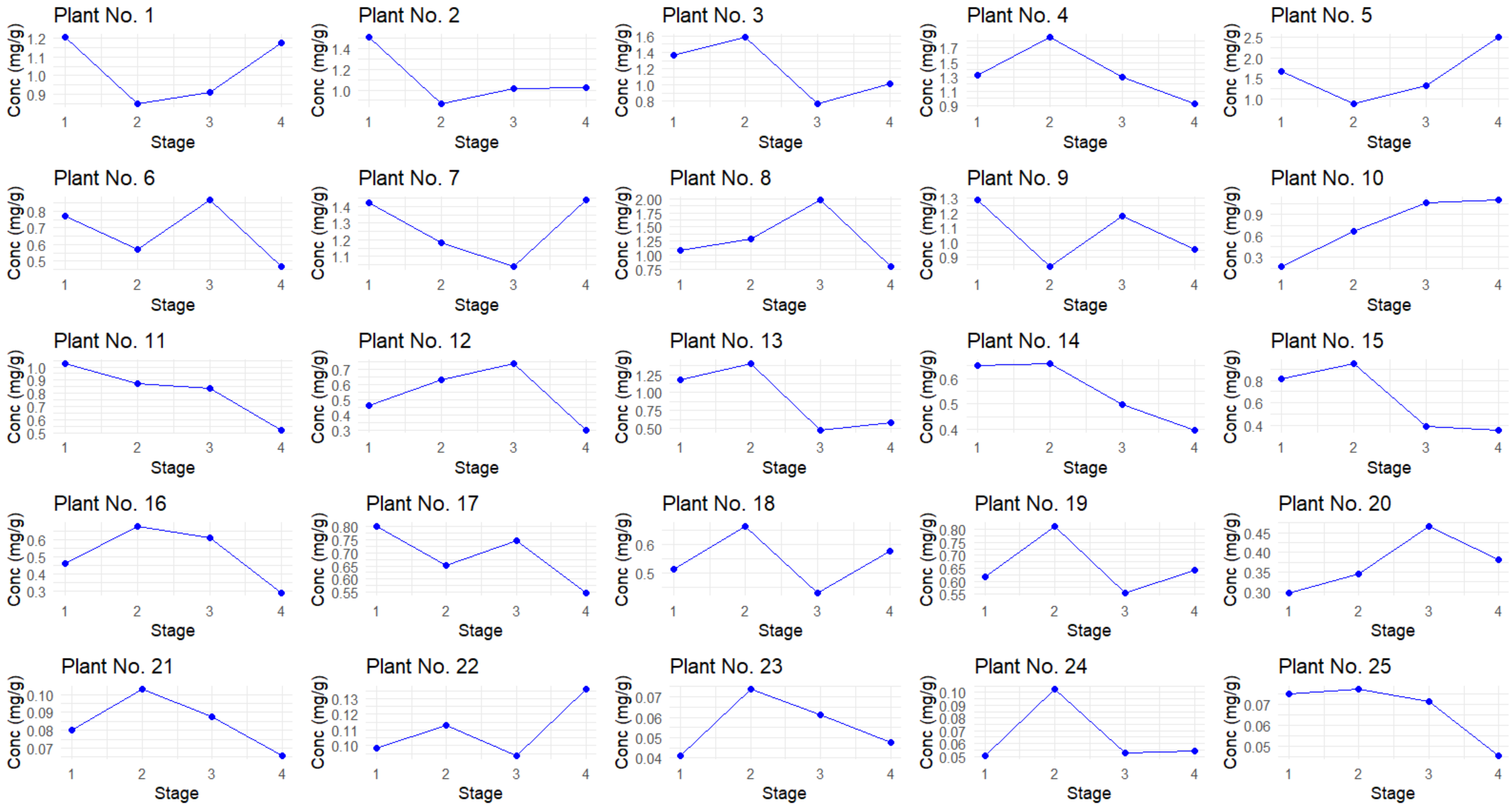

Figure S1.9 Tetrahydrocannabinol (THC) concentrations over four growth stages in 25 unique cultivars.

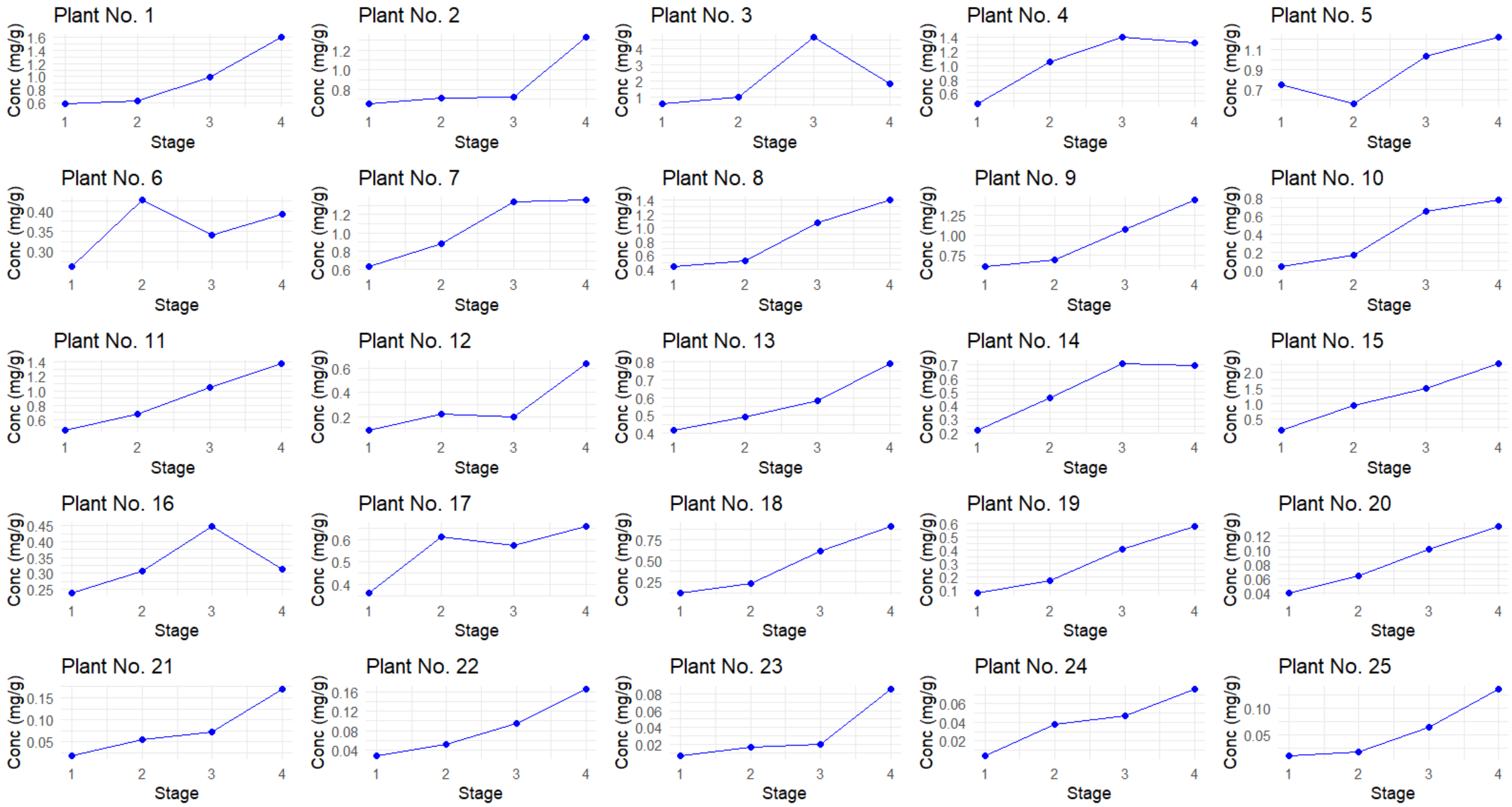

Unofficial

Figure S1.10 Tetrahydrocannabinolic Acid (THCA) concentrations over four growth stages in 25 unique cultivars.

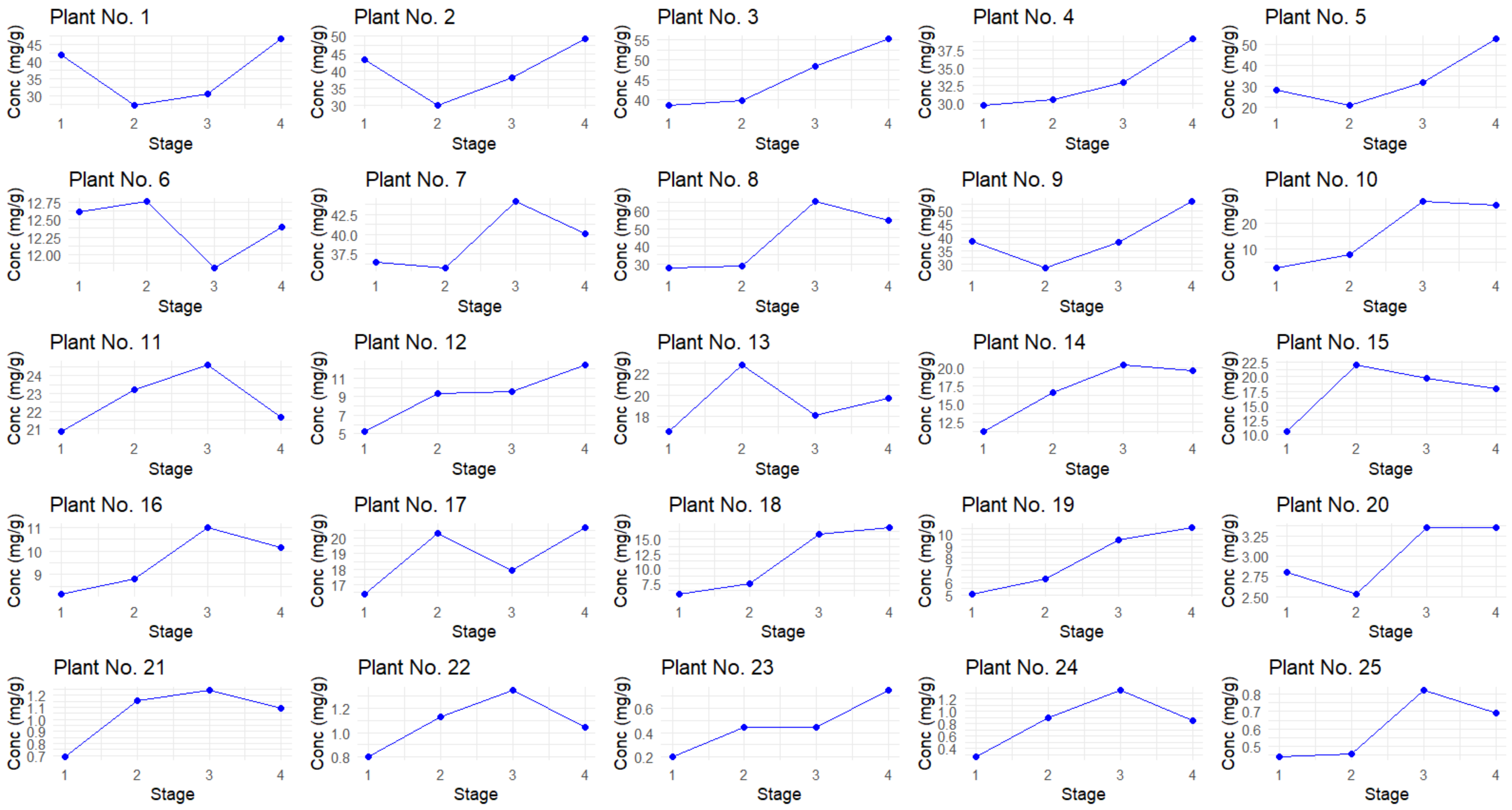

Figure S1.11 Tetrahydrocannabivarin (THCV) concentrations over four growth stages in 25 unique cultivars.

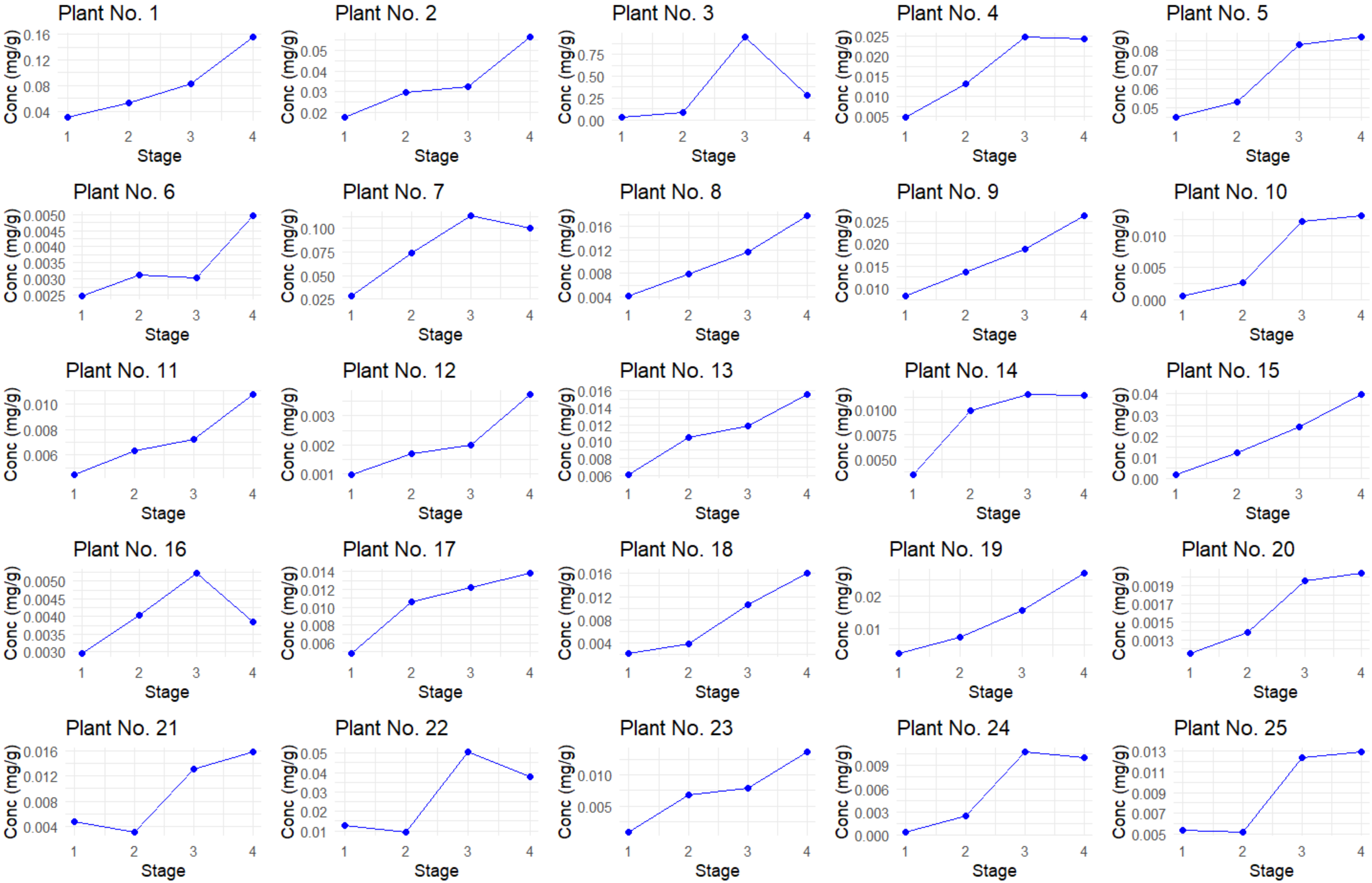

Unofficial

Figure S1.12 Tetrahydrocannabivarinic Acid (THCVA) concentrations over four growth stages in 25 unique cultivars.

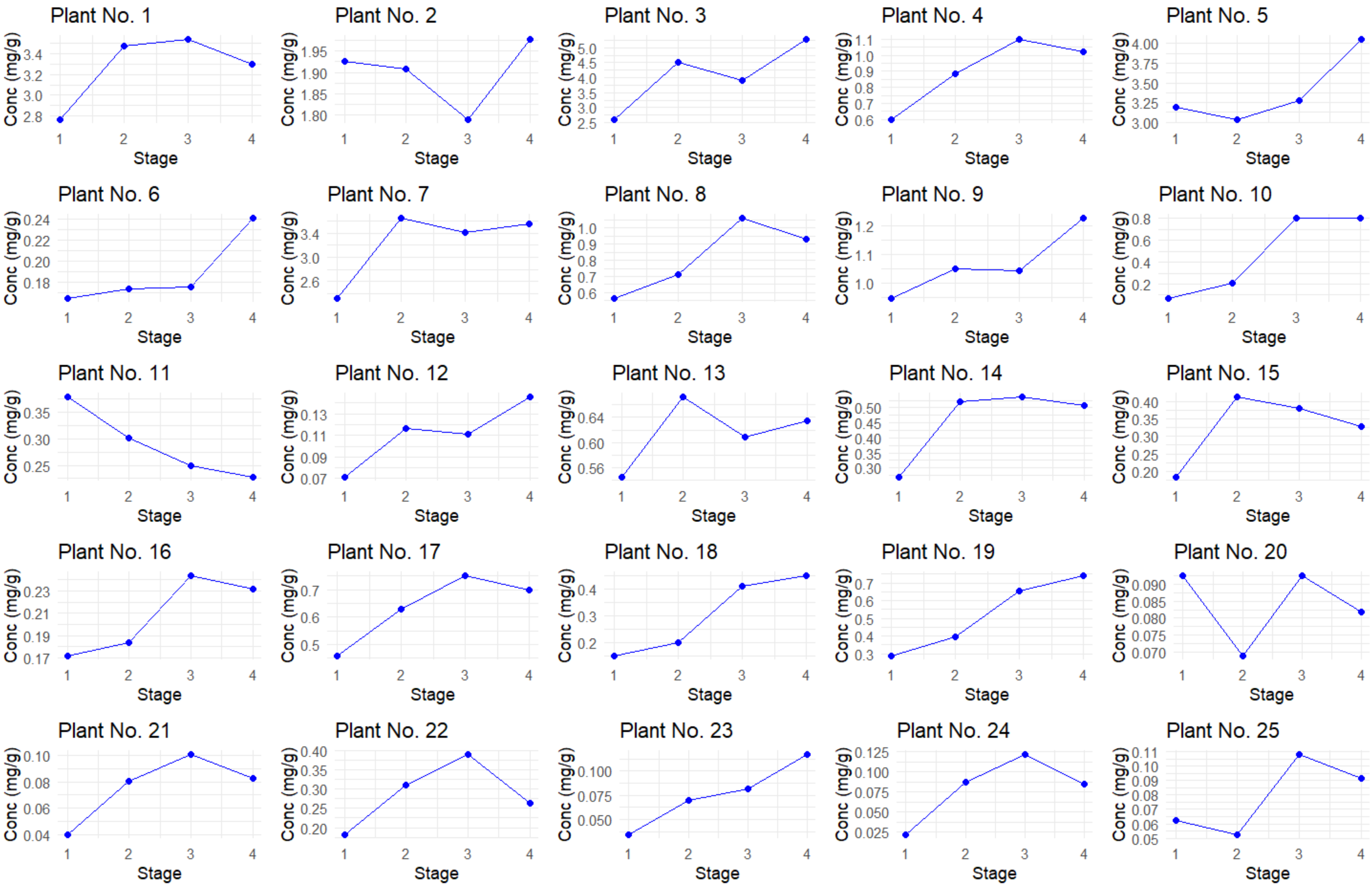

Unofficial

Figure S1.13 Cannabichromene (CBC) concentrations over four growth stages in 25 unique cultivars.

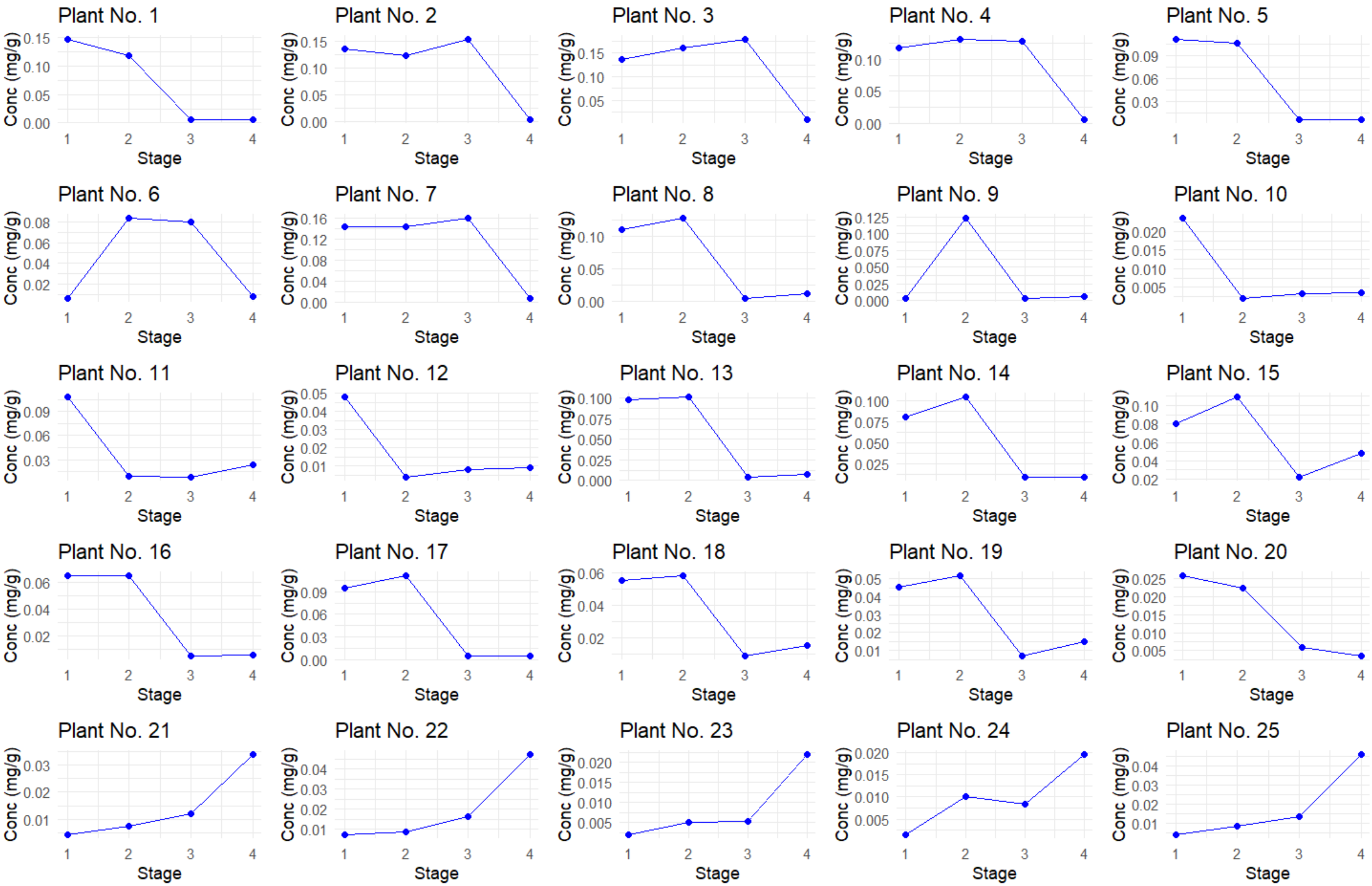

Unofficial

Figure S1.14 Cannabichromenic Acid (CBCA) concentrations over four growth stages in 25 unique cultivars.

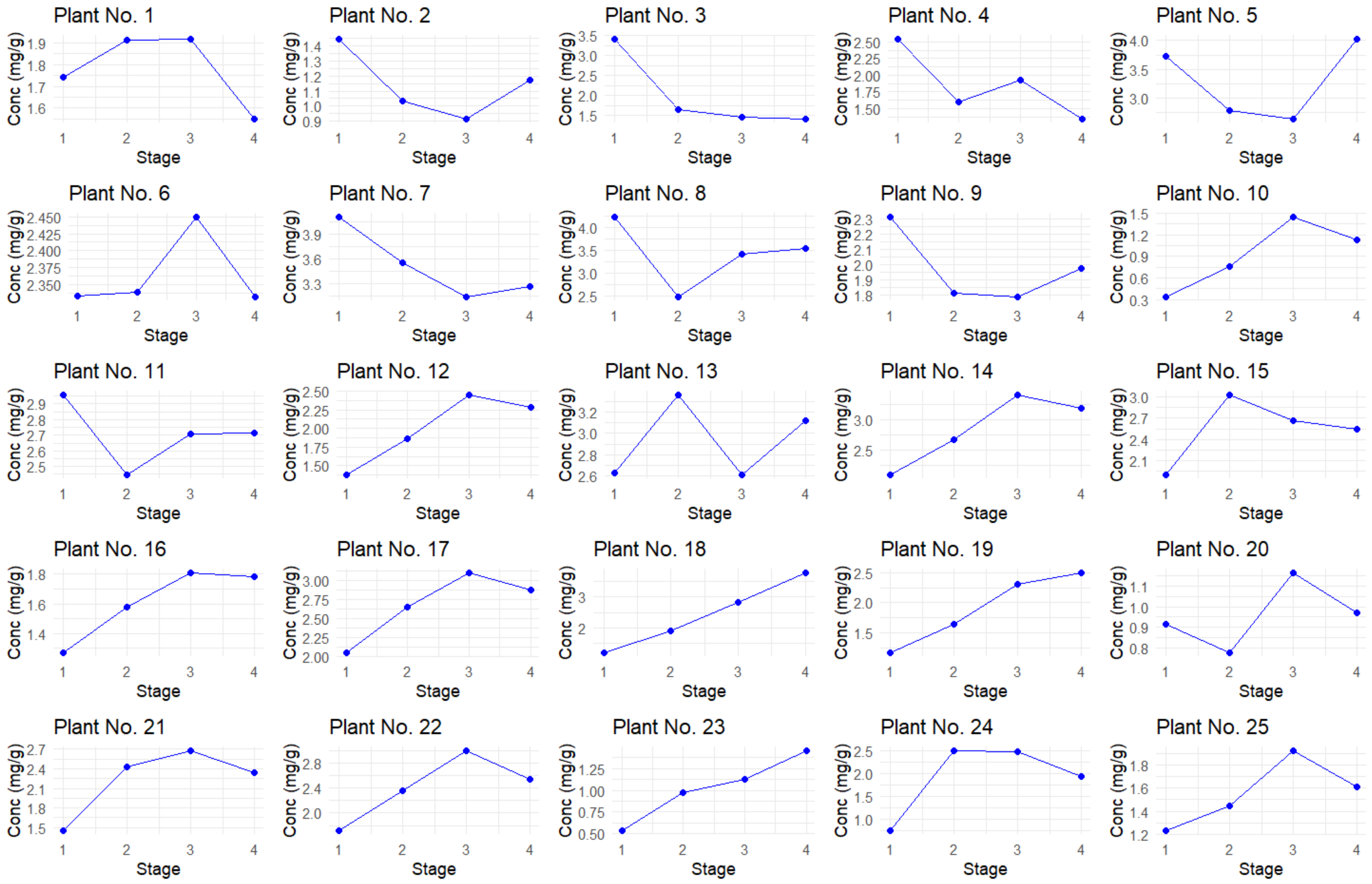

Unofficial
