# Supplementary material for: Determination of Optimal Harvest Time in Cannabis sativa L. Based upon Stigma Color Transition
Source: Plants (Basel). 2025 May 20;14(10):1532. doi: 10.3390/plants14101532 (PMC12114869; doi:10.3390/plants14101532)
Supplement: Supplementary file 1 [file plants-14-01532-s001.zip › Figure S2 The average concentration of each cannabinboid in each plant by chemovar.pdf]

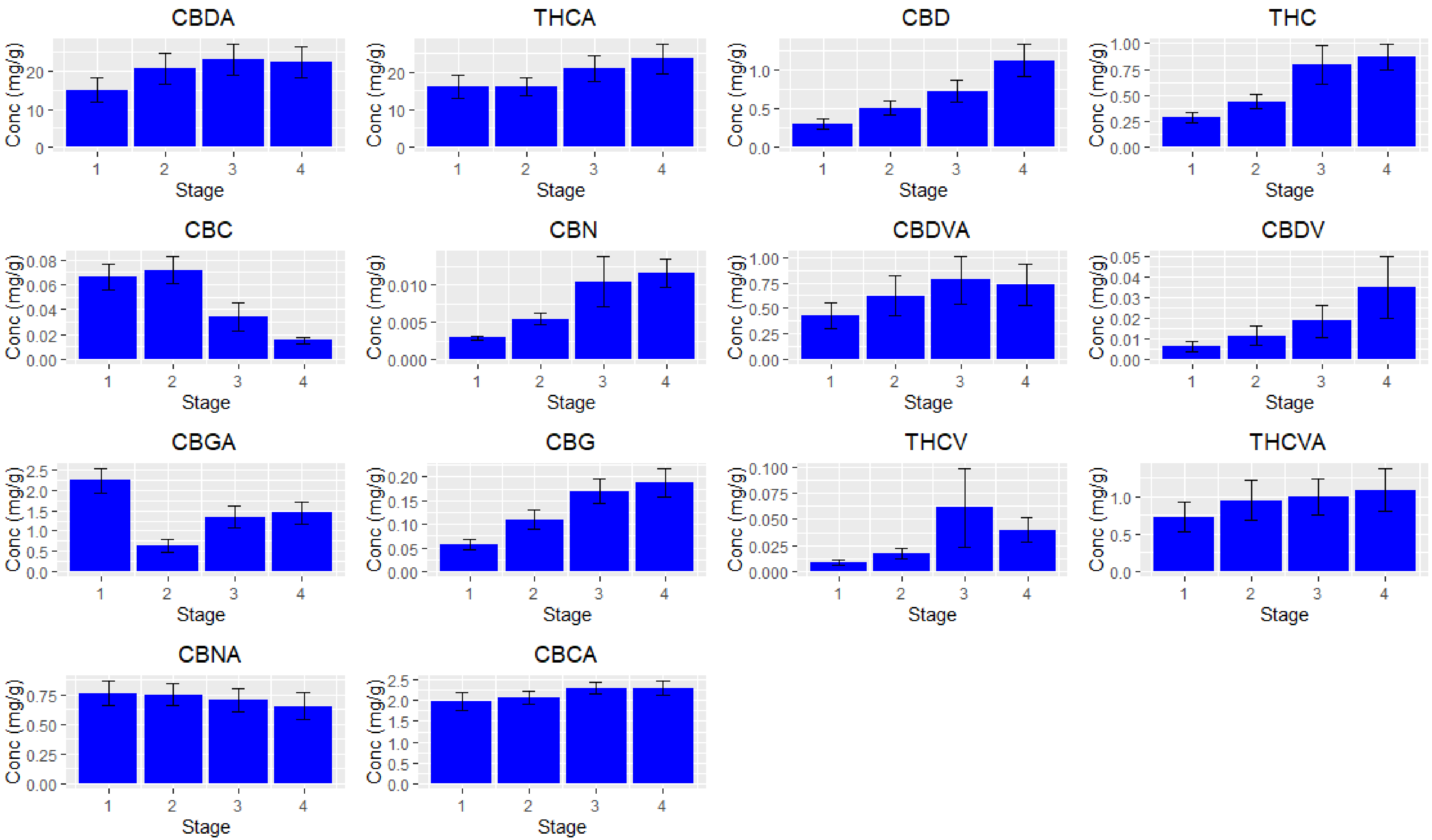

Figure S2.1. The average concentration of each cannabinoid in each plant (n = 25) over the four amber stages.

Unofficial

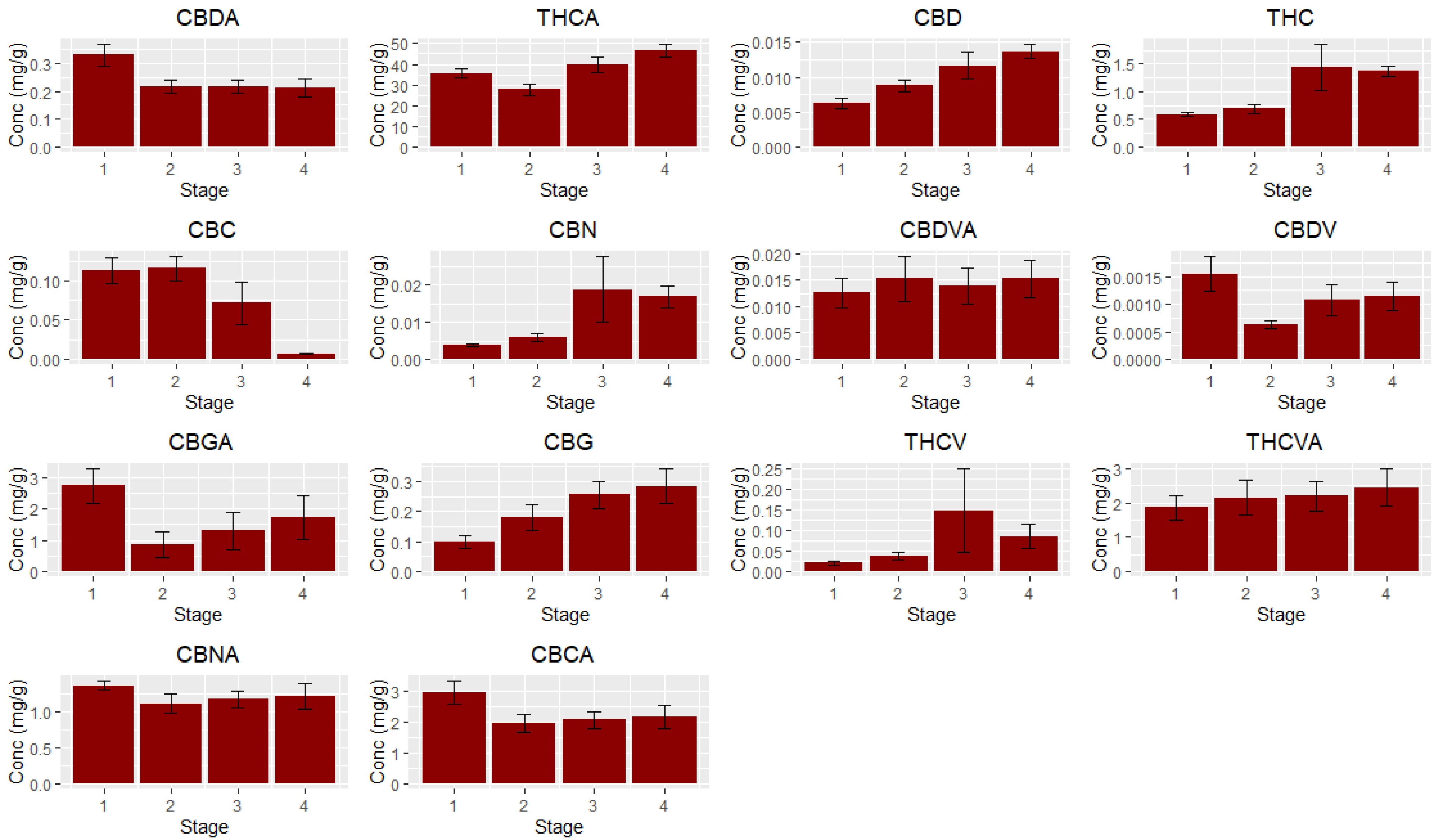

Figure S2.2. The average concentration of each cannabinoid in each high THCA plant (n = 9) over the four amber stages.

Unofficial

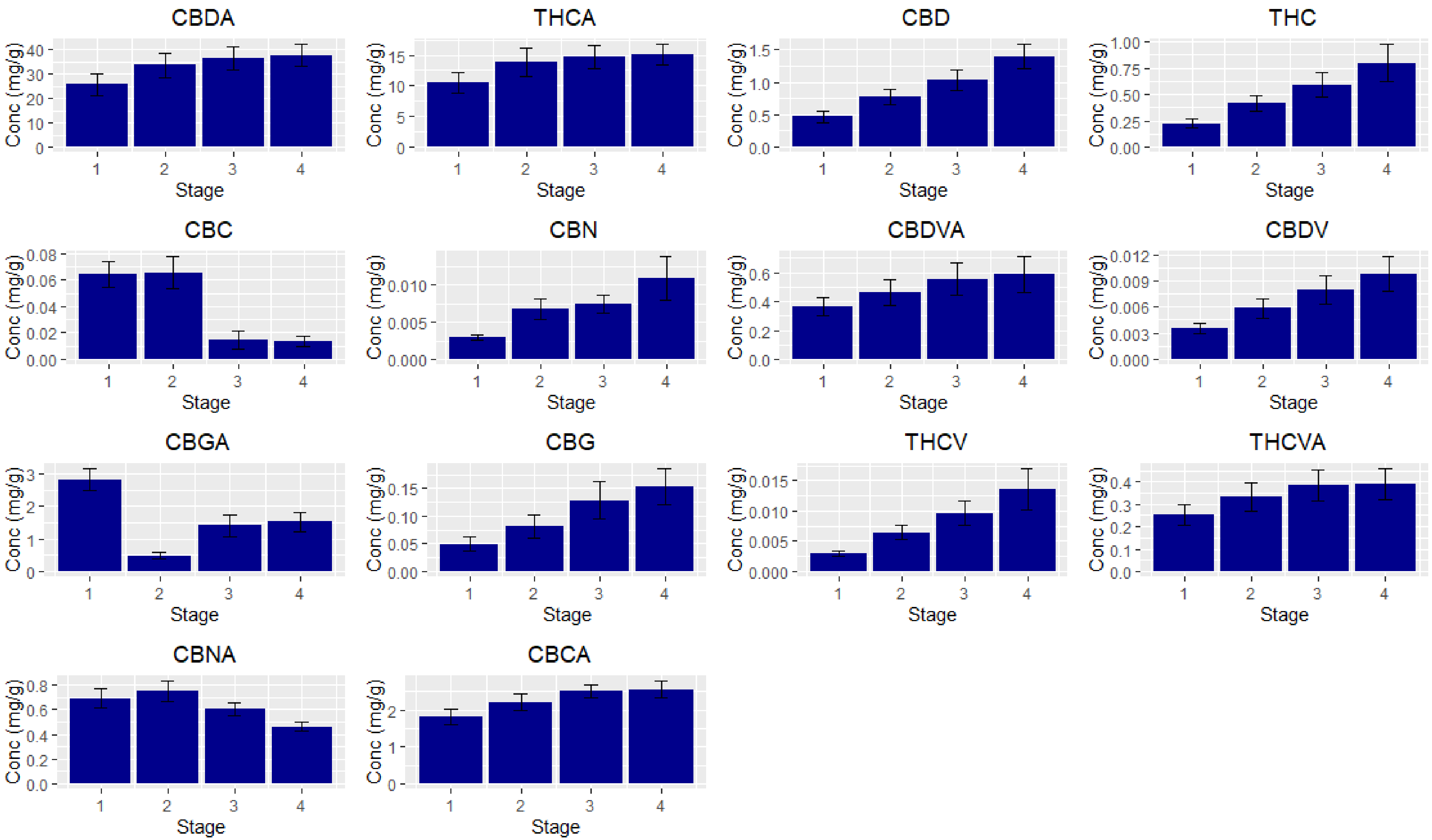

Figure S2.3. The average concentration of each cannabinoid in each even ratio plant (n = 11) over the four amber stages

Unofficial

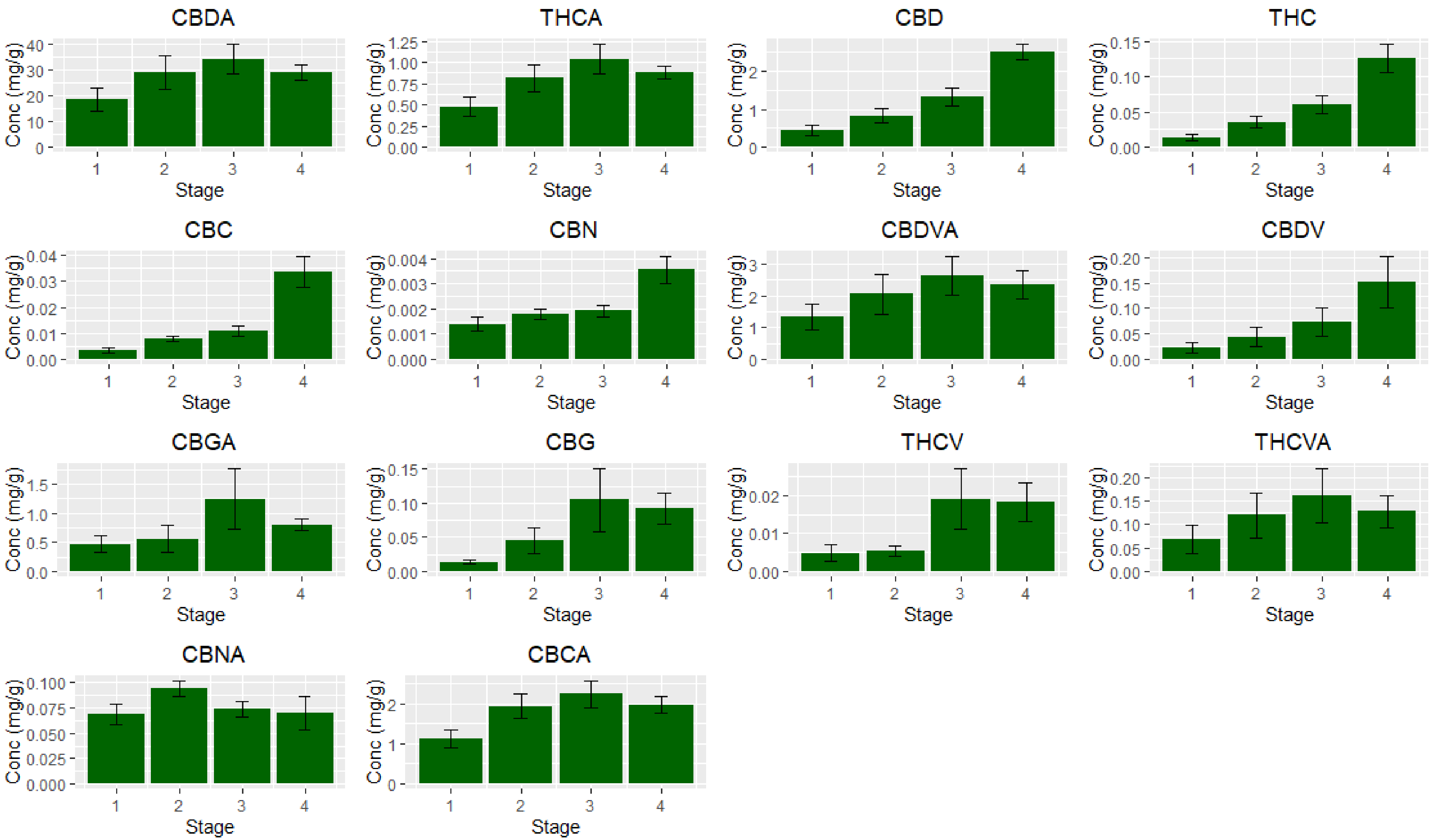

Figure S2.4. The average concentration of each cannabinoid in each high CBDA plant (n = 5) over the four amber stages

Unofficial
