# Supplementary material for: Determination of Optimal Harvest Time in Cannabis sativa L. Based upon Stigma Color Transition
Source: Plants (Basel). 2025 May 20;14(10):1532. doi: 10.3390/plants14101532 (PMC12114869; doi:10.3390/plants14101532)
Supplement: Supplementary file 1 [file plants-14-01532-s001.zip › Table and Figure S3 PLSR Permutation Results for both Full and Trim Inflorescences.pdf]

Permutation Results of PLSR models for Full Inflorescences

Probability of Model Insignificance vs. Permuted Samples
For model with 6 component(s)

Y-column: CBDA

	Wilcoxon	Sign Test	Rand t-test
Self-Prediction:	0.000	0.001	0.005
Cross-Validated:	0.000	0.000	0.005

Y-column: THCA

	Wilcoxon	Sign Test	Rand t-test
Self-Prediction:	0.000	0.001	0.005
Cross-Validated:	0.000	0.000	0.005

Y-column: CBD

	Wilcoxon	Sign Test	Rand t-test
Self-Prediction:	0.000	0.006	0.006
Cross-Validated:	0.000	0.001	0.005

Y-column: THC

	Wilcoxon	Sign Test	Rand t-test
Self-Prediction:	0.004	0.023	0.013
Cross-Validated:	0.002	0.019	0.008

Y-column: CBC

	Wilcoxon	Sign Test	Rand t-test
Self-Prediction:	0.159	0.223	0.304
Cross-Validated:	0.068	0.138	0.130

Y-column: CBN

	Wilcoxon	Sign Test	Rand t-test
Self-Prediction:	0.095	0.186	0.174
Cross-Validated:	0.056	0.127	0.118

Y-column: CBDVA

	Wilcoxon	Sign Test	Rand t-test
Self-Prediction:	0.004	0.021	0.017
Cross-Validated:	0.000	0.002	0.005

Y-column: CBDV

	Wilcoxon	Sign Test	Rand t-test
Self-Prediction:	0.331	0.365	0.316
Cross-Validated:	0.120	0.208	0.108

Y-column: CBGA

	Wilcoxon	Sign Test	Rand t-test
Self-Prediction:	0.140	0.210	0.379
Cross-Validated:	0.077	0.143	0.149

Y-column: CBG

	Wilcoxon	Sign Test	Rand t-test
Self-Prediction:	0.005	0.026	0.011
Cross-Validated:	0.004	0.027	0.007

Y-column: THCV

Unofficial

	Wilcoxon	Sign Test	Rand t-test
Self-Prediction:	0.539	0.541	0.740
Cross-Validated:	0.294	0.333	0.584

Y-column: THCVA

	Wilcoxon	Sign Test	Rand t-test
Self-Prediction:	0.037	0.098	0.028
Cross-Validated:	0.012	0.040	0.013

Y-column: CBNA

	Wilcoxon	Sign Test	Rand t-test
Self-Prediction:	0.000	0.003	0.006
Cross-Validated:	0.000	0.002	0.005

Y-column: CBCA

	Wilcoxon	Sign Test	Rand t-test
Self-Prediction:	0.002	0.020	0.040
Cross-Validated:	0.000	0.008	0.012

Values less than 0.05 indicate the model is significant at the 95% confidence level.

CBDA

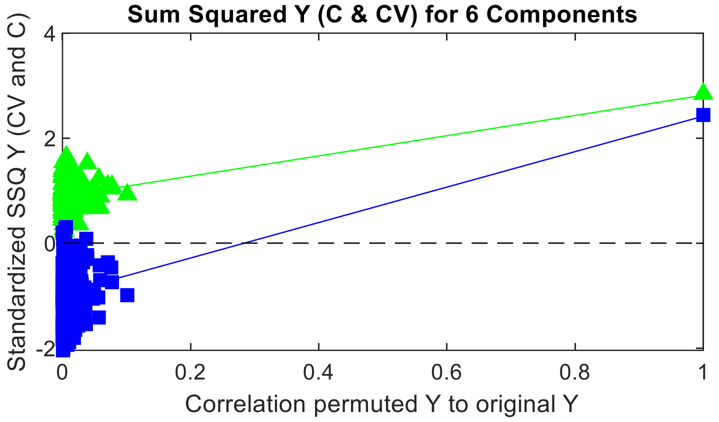

THCA

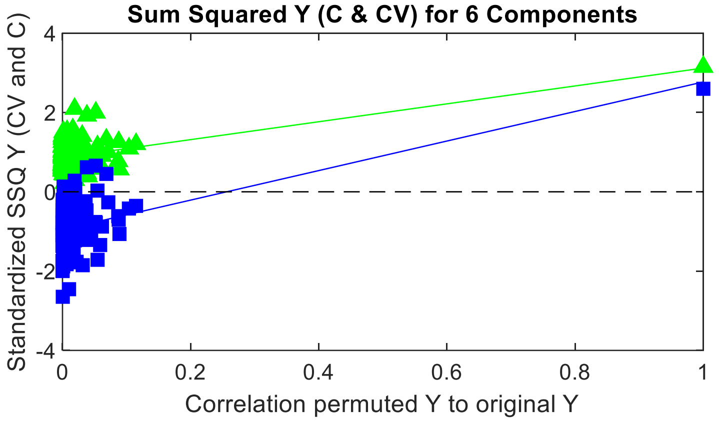

CBD

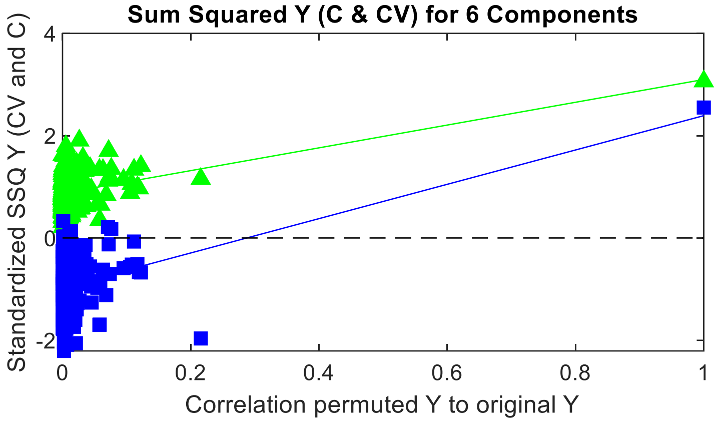

THC

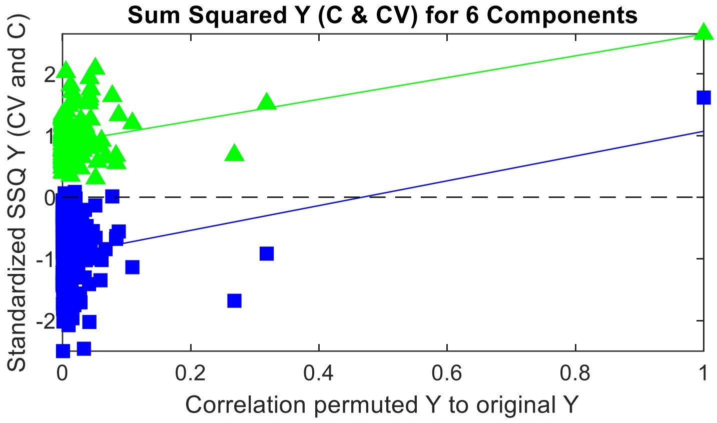

CBC

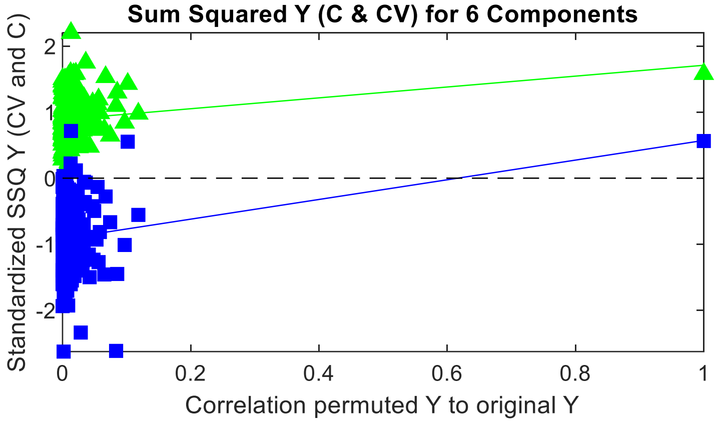

CBN

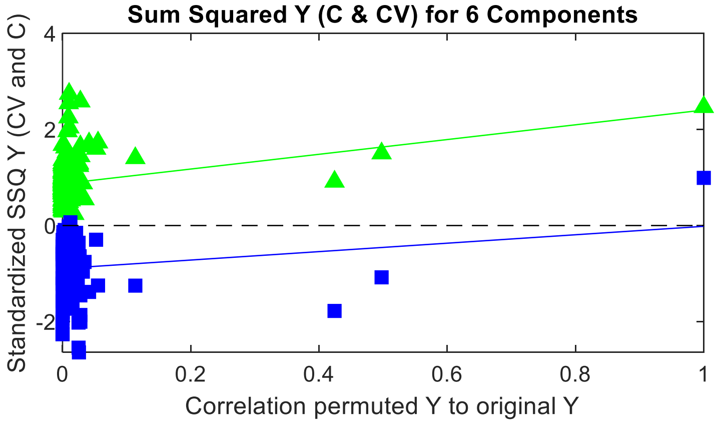

Unofficial

CBDVA

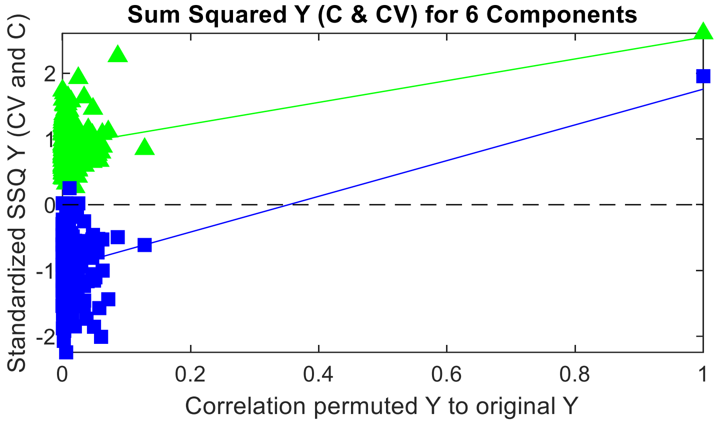

CBDV

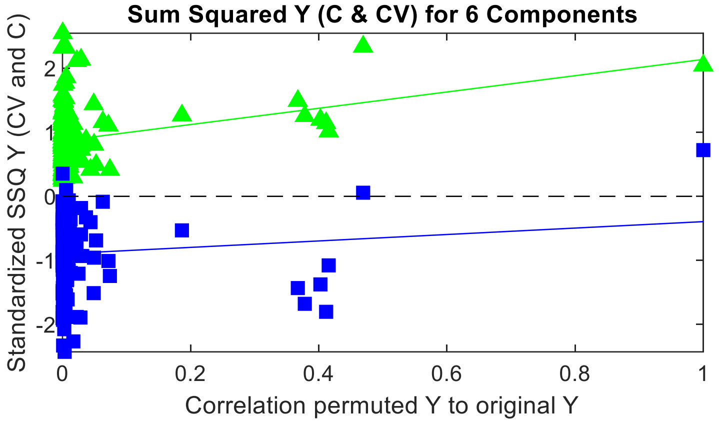

CBGA

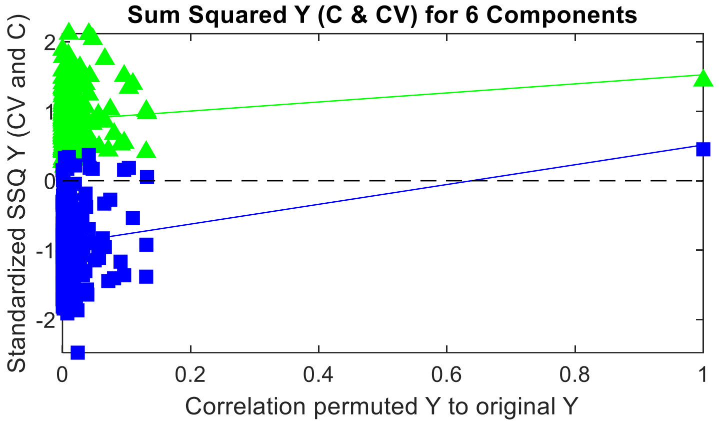

CBG

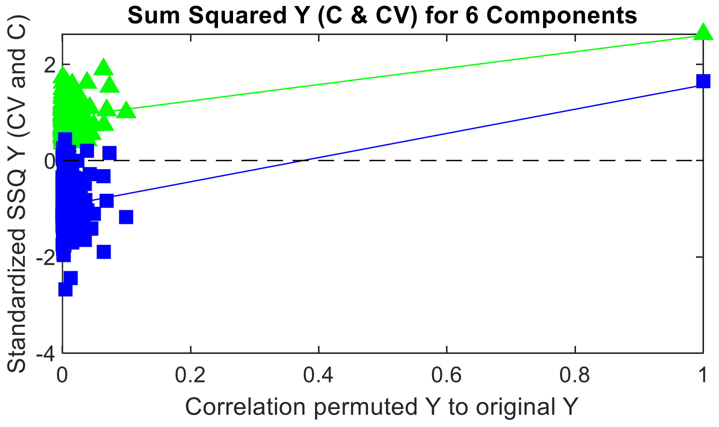

Unofficial

THCV

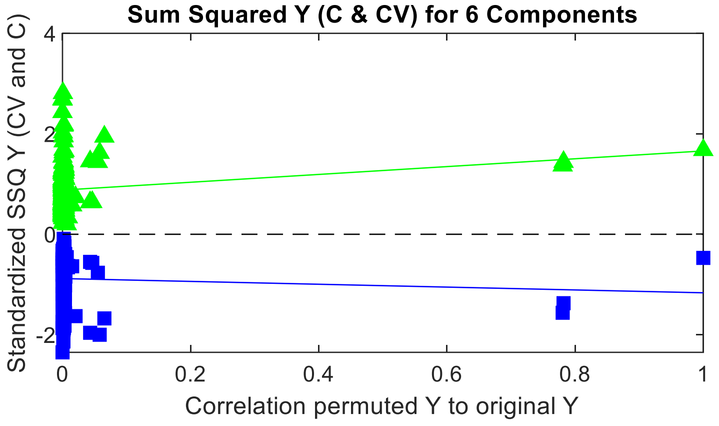

THCVA

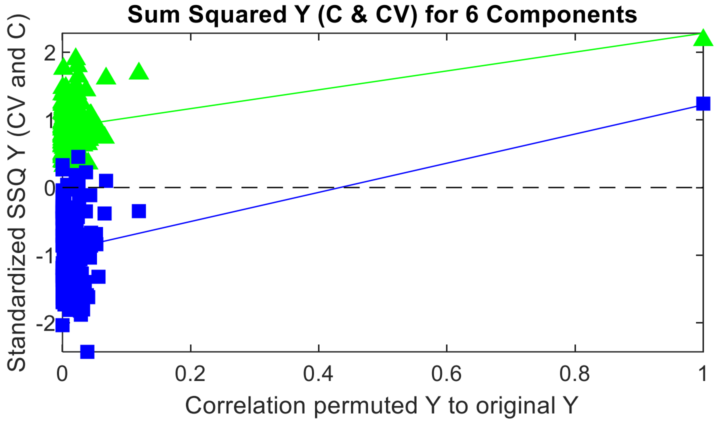

CBNA

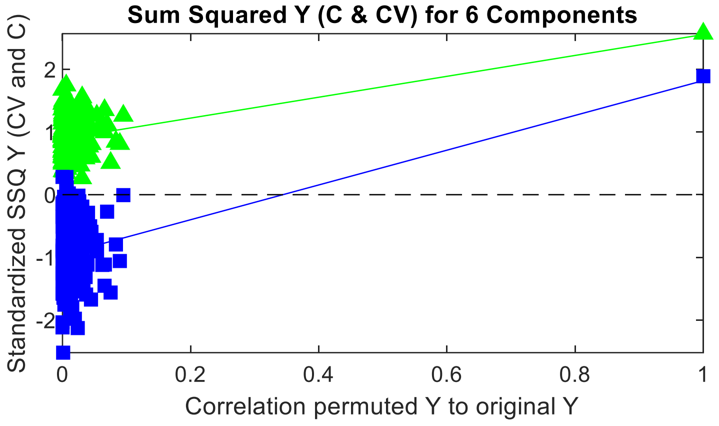

CBCA

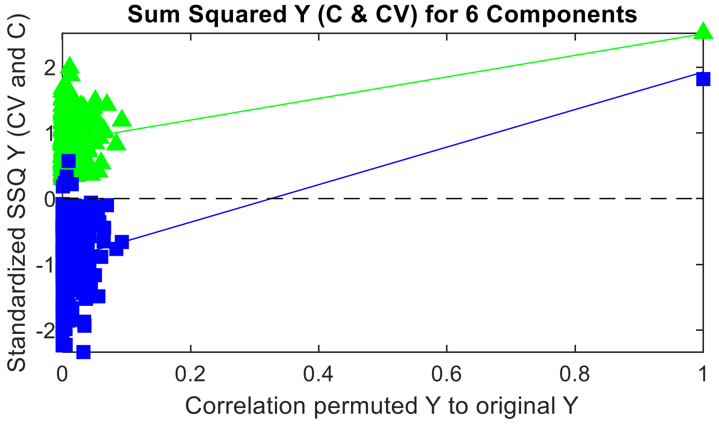

Unofficial

Permutation Results for PLSR models in Trim Inflorescences

Probability of Model Insignificance vs. Permuted Samples
For model with 8 component(s)

Y-column: CBDA

	Wilcoxon	Sign Test	Rand t-test
Self-Prediction:	0.000	0.000	0.005
Cross-Validated:	0.000	0.000	0.005

Y-column: THCA

	Wilcoxon	Sign Test	Rand t-test
Self-Prediction:	0.000	0.002	0.005
Cross-Validated:	0.000	0.000	0.005

Y-column: CBD

	Wilcoxon	Sign Test	Rand t-test
Self-Prediction:	0.000	0.001	0.005
Cross-Validated:	0.000	0.000	0.005

Y-column: THC

	Wilcoxon	Sign Test	Rand t-test
Self-Prediction:	0.006	0.044	0.054
Cross-Validated:	0.000	0.006	0.005

Y-column: CBC

	Wilcoxon	Sign Test	Rand t-test
Self-Prediction:	0.199	0.270	0.365
Cross-Validated:	0.046	0.083	0.065

Y-column: CBN

	Wilcoxon	Sign Test	Rand t-test
Self-Prediction:	0.084	0.177	0.222
Cross-Validated:	0.016	0.062	0.179

Y-column: CBDVA

	Wilcoxon	Sign Test	Rand t-test
Self-Prediction:	0.007	0.043	0.018
Cross-Validated:	0.001	0.015	0.006

Y-column: CBDV

	Wilcoxon	Sign Test	Rand t-test
Self-Prediction:	0.156	0.272	0.188
Cross-Validated:	0.088	0.190	0.068

Y-column: CBGA

	Wilcoxon	Sign Test	Rand t-test
Self-Prediction:	0.541	0.579	0.652
Cross-Validated:	0.348	0.422	0.594

Y-column: CBG

	Wilcoxon	Sign Test	Rand t-test
Self-Prediction:	0.023	0.063	0.035
Cross-Validated:	0.001	0.012	0.007

Y-column: THCV

	Wilcoxon	Sign Test	Rand t-test

Unofficial

	Wilcoxon	Sign Test	Rand t-test
Self-Prediction:	0.340	0.463	0.656
Cross-Validated:	0.102	0.194	0.899

Y-column: THCVA

	Wilcoxon	Sign Test	Rand t-test
Self-Prediction:	0.006	0.038	0.010
Cross-Validated:	0.001	0.008	0.005

Y-column: CBNA

	Wilcoxon	Sign Test	Rand t-test
Self-Prediction:	0.000	0.007	0.005
Cross-Validated:	0.000	0.001	0.005

Y-column: CBCA

	Wilcoxon	Sign Test	Rand t-test
Self-Prediction:	0.016	0.051	0.350
Cross-Validated:	0.011	0.040	0.170

Values less than 0.05 indicate the model is significant at the 95% confidence level.

CBDA

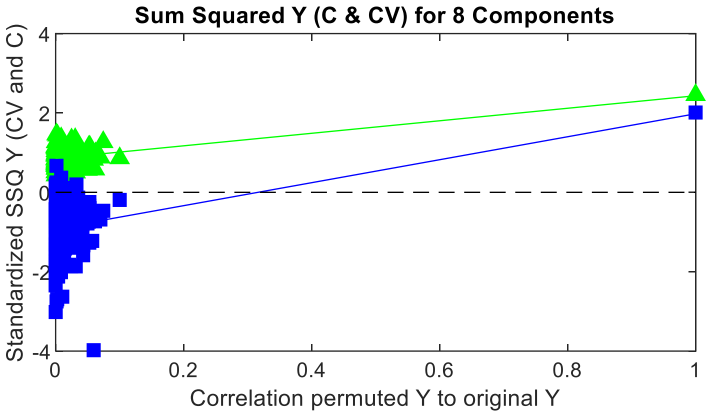

THCA

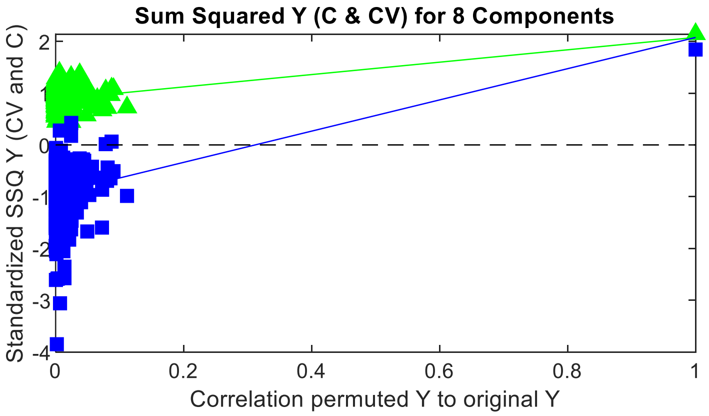

Unofficial

CBD

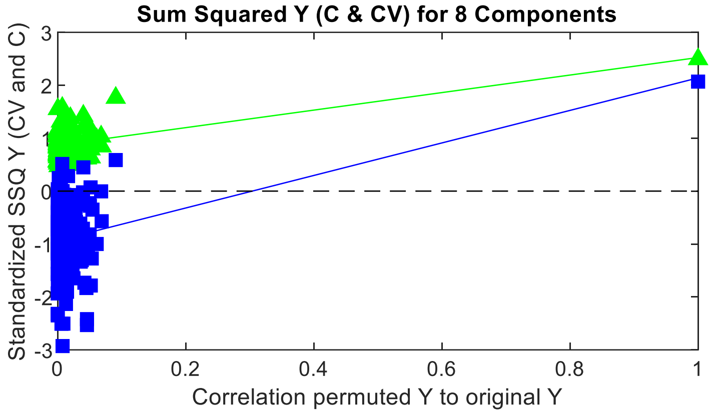

THC

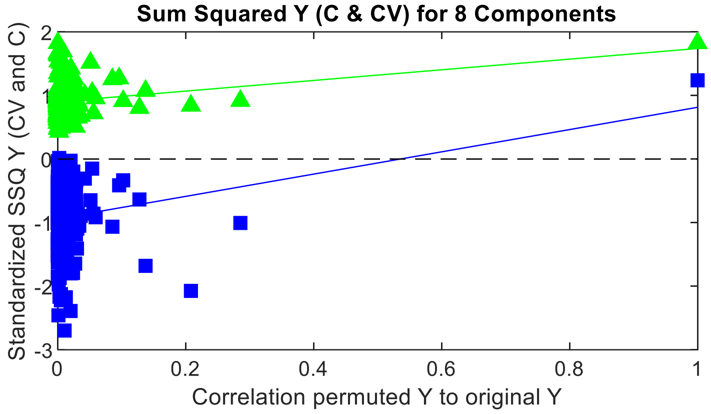

CBC

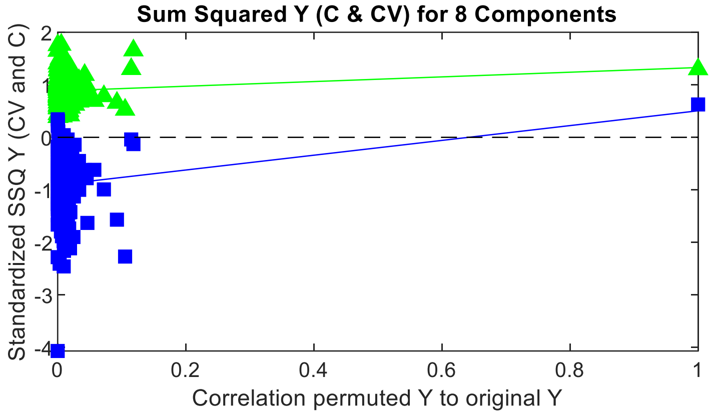

CBN

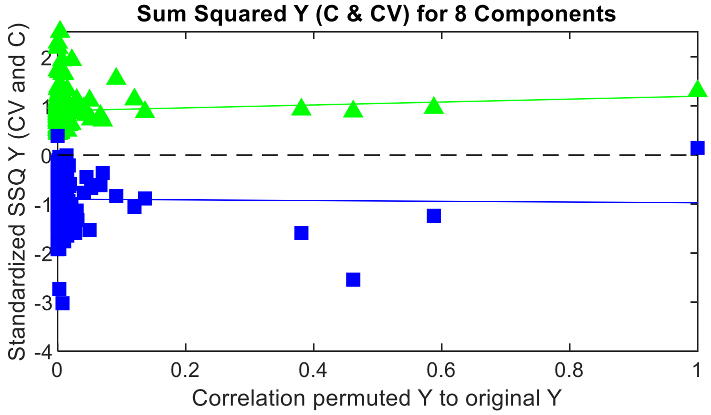

Unofficial

CBDVA

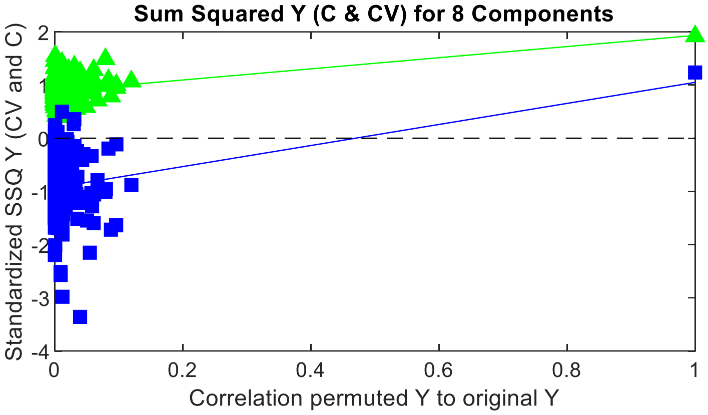

CBDV

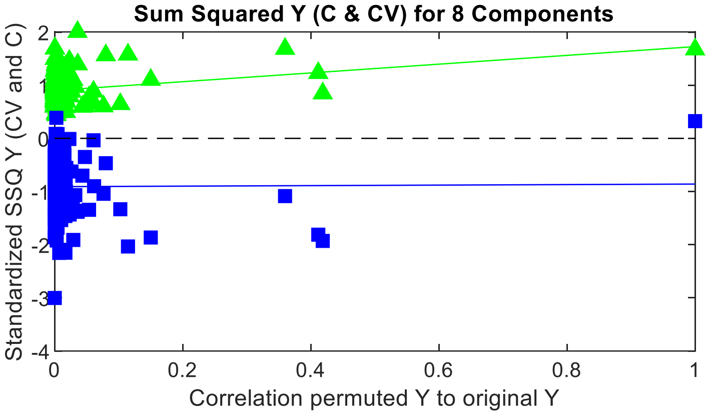

CBGA

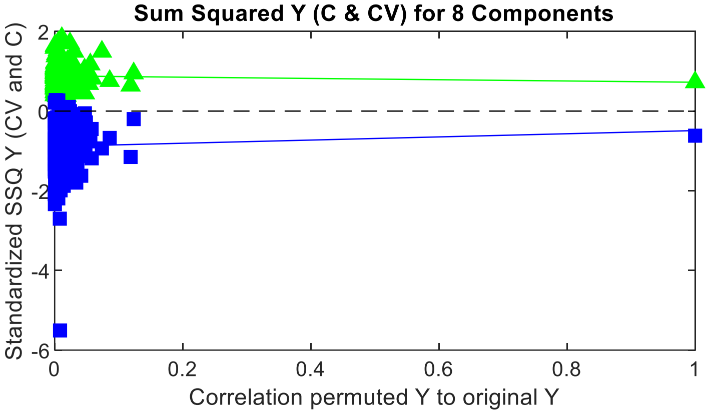

CBG

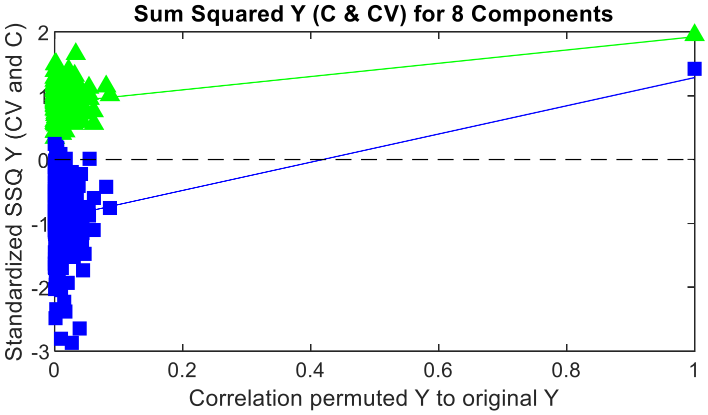

Unofficial

THCV

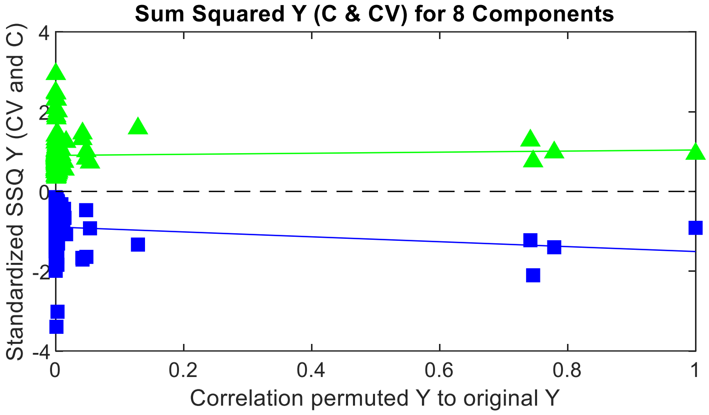

THCVA

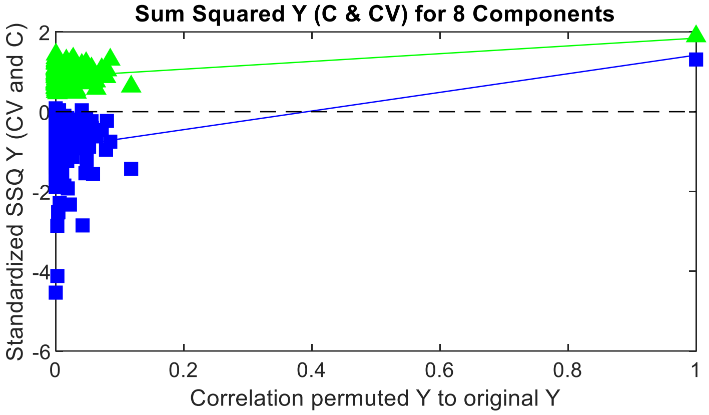

CBNA

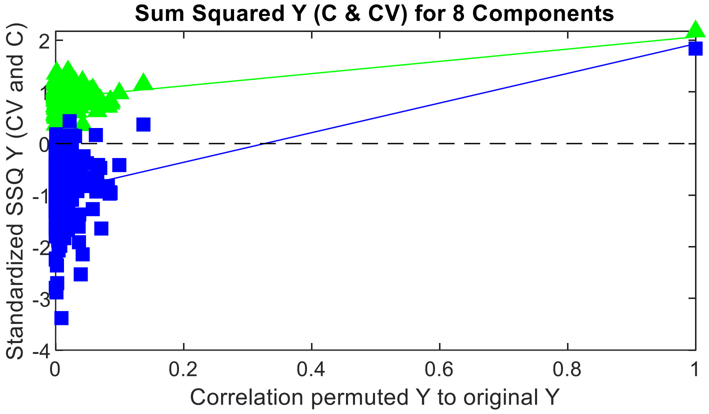

CBCA

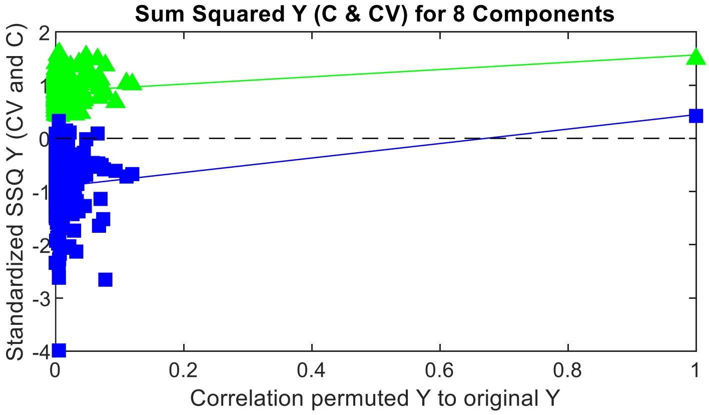

Unofficial
