# Supplementary material for: Determination of Optimal Harvest Time in Cannabis sativa L. Based upon Stigma Color Transition
Source: Plants (Basel). 2025 May 20;14(10):1532. doi: 10.3390/plants14101532 (PMC12114869; doi:10.3390/plants14101532)
Supplement: Supplementary file 1 [file plants-14-01532-s001.zip › Table S1 LCMS Cannabinoid Data.pdf]

Table S1. Liquid Chromatography Mass Spectroscopy data for all cannabinoids analysed in the harvest optimisation trial (n = 100)

		Concentration (mg/g)														
Stage	Genotype	CBDA	THCA	CBD	THC	CBC	CBN	CBDVA	CBDV	CBGA	CBG	THCV	THCVA	CBNA	CBCA	Total
1	1	0.33	41.95	0.01	0.58	0.15	0.00	0.02	0.00	1.04	0.09	0.03	2.76	1.20	1.74	49.91
1	2	0.35	43.23	0.00	0.65	0.14	0.00	0.01	0.00	0.38	0.05	0.02	1.93	1.51	1.45	49.72
1	3	0.16	38.59	0.01	0.56	0.14	0.00	0.02	0.00	5.00	0.15	0.03	2.59	1.37	3.41	52.01
1	4	0.27	29.64	0.01	0.45	0.12	0.00	0.00	0.00	3.11	0.04	0.00	0.60	1.32	2.54	38.11
1	5	0.54	28.37	0.01	0.75	0.11	0.01	0.02	0.00	3.22	0.21	0.05	3.19	1.67	3.73	41.89
1	6	35.81	12.62	0.72	0.26	0.01	0.00	0.27	0.00	3.55	0.05	0.00	0.16	0.77	2.33	56.56
1	7	0.38	36.47	0.01	0.63	0.14	0.00	0.02	0.00	3.83	0.06	0.03	2.31	1.43	4.11	49.42
1	8	0.34	27.45	0.00	0.43	0.11	0.00	0.01	0.00	3.50	0.12	0.00	0.56	1.09	4.22	37.85
1	9	0.28	38.44	0.00	0.60	0.00	0.00	0.00	0.00	1.73	0.07	0.01	0.95	1.28	2.31	45.69
1	10	0.03	2.56	0.01	0.03	0.02	0.00	0.00	0.00	0.80	0.00	0.00	0.07	0.17	0.33	4.03
1	11	48.34	20.87	0.87	0.46	0.11	0.00	0.41	0.00	2.96	0.14	0.00	0.38	1.03	2.96	78.54
1	12	12.09	5.26	0.19	0.09	0.05	0.00	0.11	0.00	1.86	0.01	0.00	0.07	0.46	1.37	21.59
1	13	48.20	16.57	0.96	0.42	0.10	0.00	0.82	0.01	4.16	0.08	0.01	0.54	1.18	2.63	75.68
1	14	28.75	11.28	0.55	0.22	0.08	0.00	0.43	0.00	3.97	0.07	0.00	0.27	0.65	2.08	48.36
1	15	18.97	10.51	0.27	0.16	0.08	0.00	0.23	0.00	2.54	0.02	0.00	0.18	0.81	1.90	35.68
1	16	16.26	8.17	0.33	0.24	0.07	0.00	0.24	0.00	0.99	0.02	0.00	0.17	0.46	1.27	28.21
1	17	37.25	16.41	0.73	0.36	0.09	0.00	0.59	0.01	4.33	0.09	0.00	0.46	0.80	2.05	63.18
1	18	15.52	5.70	0.27	0.12	0.06	0.00	0.28	0.00	2.49	0.02	0.00	0.15	0.52	1.19	26.31
1	19	13.56	5.05	0.19	0.08	0.05	0.00	0.50	0.00	2.72	0.02	0.00	0.29	0.62	1.16	24.23
1	20	7.60	2.80	0.10	0.04	0.03	0.00	0.15	0.00	1.30	0.01	0.00	0.09	0.30	0.91	13.32
1	21	24.73	0.69	0.64	0.02	0.00	0.00	0.85	0.01	0.19	0.01	0.00	0.04	0.08	1.45	28.72
1	22	32.78	0.80	0.83	0.03	0.01	0.00	2.82	0.06	0.25	0.02	0.01	0.18	0.10	1.71	39.60
1	23	8.09	0.20	0.18	0.01	0.00	0.00	1.00	0.01	0.25	0.00	0.00	0.03	0.04	0.54	10.35
1	24	9.61	0.26	0.18	0.00	0.00	0.00	0.62	0.01	0.91	0.01	0.00	0.02	0.05	0.74	12.41
1	25	16.88	0.44	0.37	0.01	0.00	0.00	1.40	0.02	0.79	0.02	0.01	0.06	0.07	1.23	21.31
2	1	0.24	27.01	0.01	0.62	0.12	0.00	0.03	0.00	0.10	0.18	0.05	3.48	0.85	1.91	34.60
2	2	0.21	30.02	0.01	0.71	0.12	0.00	0.01	0.00	0.07	0.12	0.03	1.91	0.87	1.03	35.11
2	3	0.21	39.78	0.01	0.97	0.16	0.01	0.04	0.00	0.96	0.22	0.08	4.51	1.58	1.64	50.18
2	4	0.20	30.44	0.01	1.05	0.13	0.01	0.00	0.00	3.96	0.15	0.01	0.89	1.85	1.60	40.29
2	5	0.31	20.81	0.01	0.56	0.11	0.00	0.03	0.00	0.33	0.46	0.05	3.03	0.88	2.79	29.37
2	6	41.04	12.75	1.10	0.43	0.08	0.00	0.27	0.00	0.10	0.09	0.00	0.17	0.57	2.34	58.95
2	7	0.24	35.67	0.01	0.88	0.14	0.01	0.02	0.00	0.41	0.10	0.07	3.65	1.18	3.55	45.95
2	8	0.28	29.03	0.01	0.52	0.13	0.00	0.00	0.00	0.88	0.15	0.01	0.71	1.29	2.47	35.49
2	9	0.19	28.34	0.01	0.69	0.12	0.00	0.01	0.00	0.18	0.24	0.01	1.05	0.84	1.81	33.50
2	10	0.07	8.02	0.01	0.17	0.00	0.00	0.00	0.00	0.88	0.01	0.00	0.21	0.66	0.76	10.79
2	11	45.50	23.23	1.10	0.68	0.01	0.01	0.30	0.00	0.20	0.19	0.01	0.30	0.88	2.45	74.85
2	12	23.73	9.35	0.49	0.22	0.00	0.00	0.17	0.00	0.30	0.01	0.00	0.12	0.63	1.86	36.90
2	13	55.95	22.88	0.98	0.49	0.10	0.01	0.80	0.01	0.26	0.11	0.01	0.67	1.41	3.36	87.06
2	14	55.22	16.61	1.33	0.46	0.10	0.01	0.87	0.01	1.31	0.18	0.01	0.52	0.66	2.67	79.97

Unofficial

2	15	36.36	22.12	0.72	0.93	0.11	0.02	0.39	0.00	0.25	0.09	0.01	0.41	0.95	3.03	65.40
2	16	17.83	8.79	0.46	0.31	0.07	0.01	0.24	0.00	0.28	0.02	0.00	0.18	0.68	1.58	30.44
2	17	46.25	20.28	1.14	0.61	0.11	0.01	0.73	0.01	0.51	0.14	0.01	0.63	0.65	2.65	73.71
2	18	23.32	7.48	0.59	0.23	0.06	0.01	0.38	0.00	0.79	0.03	0.00	0.20	0.67	1.89	35.66
2	19	18.54	6.33	0.44	0.17	0.05	0.00	0.83	0.01	0.70	0.02	0.01	0.39	0.81	1.63	29.94
2	20	7.24	2.54	0.17	0.06	0.02	0.00	0.13	0.00	0.68	0.01	0.00	0.07	0.34	0.78	12.05
2	21	43.31	1.15	1.24	0.06	0.01	0.00	1.49	0.03	0.28	0.04	0.00	0.08	0.10	2.43	50.22
2	22	41.85	1.13	1.20	0.05	0.01	0.00	4.52	0.12	0.26	0.04	0.01	0.31	0.11	2.35	51.96
2	23	14.32	0.44	0.41	0.02	0.00	0.00	1.32	0.03	0.19	0.01	0.01	0.07	0.07	0.97	17.86
2	24	32.87	0.89	0.91	0.04	0.01	0.00	1.68	0.03	1.45	0.12	0.00	0.09	0.10	2.51	40.70
2	25	13.33	0.46	0.34	0.02	0.01	0.00	1.27	0.02	0.62	0.02	0.01	0.05	0.08	1.45	17.67
3	1	0.23	30.35	0.01	1.00	0.01	0.01	0.02	0.00	0.07	0.20	0.08	3.54	0.91	1.92	38.34
3	2	0.18	37.99	0.01	0.72	0.15	0.00	0.01	0.00	0.06	0.12	0.03	1.79	1.01	0.91	42.99
3	3	0.20	48.26	0.02	4.67	0.18	0.09	0.03	0.00	3.17	0.31	0.95	3.89	0.77	1.45	64.00
3	4	0.25	32.86	0.01	1.40	0.13	0.02	0.01	0.00	4.55	0.30	0.02	1.10	1.30	1.93	43.88
3	5	0.33	32.08	0.01	1.03	0.01	0.01	0.02	0.00	0.16	0.44	0.08	3.27	1.31	2.64	41.40
3	6	42.13	11.80	0.96	0.34	0.08	0.01	0.27	0.00	0.10	0.06	0.00	0.18	0.86	2.45	59.24
3	7	0.27	44.10	0.01	1.34	0.16	0.01	0.02	0.00	0.11	0.15	0.11	3.41	1.03	3.13	53.88
3	8	0.20	65.19	0.01	1.07	0.00	0.01	0.00	0.00	0.29	0.44	0.01	1.06	1.97	3.40	73.68
3	9	0.22	38.19	0.01	1.08	0.00	0.01	0.00	0.00	0.14	0.26	0.02	1.04	1.18	1.79	43.95
3	10	0.08	28.23	0.00	0.65	0.00	0.01	0.00	0.00	3.18	0.07	0.01	0.80	1.07	1.45	35.56
3	11	41.00	24.63	1.39	1.05	0.01	0.01	0.22	0.00	0.28	0.22	0.01	0.25	0.84	2.71	72.62
3	12	20.59	9.59	0.21	0.19	0.01	0.01	0.14	0.00	0.25	0.00	0.00	0.11	0.74	2.45	34.30
3	13	46.28	18.06	1.33	0.58	0.00	0.00	0.82	0.01	2.83	0.21	0.01	0.61	0.48	2.61	73.83
3	14	54.42	20.50	1.44	0.71	0.01	0.01	0.80	0.01	2.63	0.35	0.01	0.54	0.50	3.42	85.34
3	15	31.29	19.76	1.69	1.49	0.02	0.02	0.40	0.01	1.12	0.17	0.02	0.38	0.39	2.67	59.42
3	16	23.03	11.00	0.65	0.45	0.00	0.01	0.31	0.00	1.55	0.03	0.01	0.24	0.61	1.81	39.72
3	17	54.78	17.90	1.17	0.57	0.01	0.00	0.98	0.01	0.97	0.13	0.01	0.75	0.75	3.10	81.14
3	18	50.50	15.72	1.49	0.61	0.01	0.01	0.79	0.01	2.64	0.19	0.01	0.41	0.43	2.84	75.67
3	19	28.39	9.51	0.77	0.41	0.01	0.00	1.19	0.02	2.39	0.05	0.02	0.66	0.55	2.30	46.26
3	20	8.82	3.35	0.23	0.10	0.01	0.00	0.16	0.00	0.62	0.01	0.00	0.09	0.47	1.16	15.03
3	21	43.76	1.24	1.54	0.07	0.01	0.00	1.98	0.04	0.53	0.06	0.01	0.10	0.09	2.68	52.12
3	22	43.01	1.34	1.81	0.09	0.02	0.00	5.02	0.18	0.66	0.09	0.05	0.39	0.09	3.00	55.76
3	23	14.74	0.44	0.42	0.02	0.01	0.00	1.66	0.03	0.42	0.01	0.01	0.08	0.06	1.13	19.04
3	24	42.14	1.34	1.48	0.05	0.01	0.00	2.40	0.04	3.19	0.28	0.01	0.12	0.05	2.49	53.61
3	25	27.41	0.82	1.40	0.06	0.01	0.00	2.15	0.08	1.43	0.08	0.01	0.11	0.07	1.93	35.57
4	1	0.23	46.64	0.01	1.60	0.01	0.02	0.02	0.00	0.07	0.17	0.16	3.30	1.17	1.55	54.93
4	2	0.19	49.23	0.01	1.33	0.00	0.01	0.01	0.00	0.09	0.19	0.06	1.98	1.02	1.17	55.30
4	3	0.18	55.18	0.02	1.83	0.01	0.03	0.03	0.00	2.65	0.35	0.28	5.28	1.02	1.41	68.25
4	4	0.17	39.11	0.01	1.33	0.01	0.01	0.01	0.00	6.02	0.24	0.02	1.02	0.92	1.34	50.22
4	5	0.43	52.84	0.01	1.22	0.01	0.03	0.02	0.00	0.28	0.27	0.09	4.05	2.50	4.02	65.78
4	6	50.61	12.39	1.42	0.39	0.01	0.00	0.40	0.01	0.13	0.11	0.00	0.24	0.47	2.33	68.51
4	7	0.22	40.09	0.02	1.36	0.01	0.02	0.03	0.00	0.34	0.18	0.10	3.55	1.44	3.26	50.61
4	8	0.18	54.59	0.01	1.39	0.01	0.01	0.00	0.00	2.91	0.67	0.02	0.93	0.79	3.54	65.04

Unofficial

4	9	0.24	53.35	0.02	1.44	0.01	0.01	0.01	0.00	0.22	0.41	0.03	1.23	0.95	1.97	59.88
4	10	0.07	26.92	0.01	0.78	0.00	0.01	0.00	0.00	2.89	0.08	0.01	0.80	1.10	1.13	33.82
4	11	35.01	21.64	1.85	1.38	0.02	0.03	0.21	0.01	0.80	0.23	0.01	0.23	0.52	2.71	64.65
4	12	29.72	12.49	1.35	0.64	0.01	0.01	0.21	0.00	1.67	0.05	0.00	0.15	0.30	2.28	48.88
4	13	48.91	19.68	1.53	0.79	0.01	0.01	0.81	0.01	2.10	0.23	0.02	0.63	0.58	3.12	78.43
4	14	52.61	19.66	1.63	0.69	0.01	0.01	0.79	0.01	2.97	0.37	0.01	0.51	0.40	3.20	82.87
4	15	28.63	17.97	2.51	2.29	0.05	0.02	0.35	0.01	1.25	0.15	0.04	0.33	0.36	2.54	56.49
4	16	21.01	10.15	0.54	0.31	0.01	0.00	0.30	0.00	1.60	0.06	0.00	0.23	0.29	1.78	36.28
4	17	50.76	20.67	1.41	0.66	0.00	0.01	0.92	0.01	2.89	0.22	0.01	0.70	0.55	2.88	81.71
4	18	55.76	16.94	1.87	0.91	0.02	0.01	0.85	0.02	2.20	0.20	0.02	0.45	0.58	3.78	83.58
4	19	33.09	10.56	1.02	0.58	0.02	0.01	1.48	0.02	0.62	0.05	0.03	0.74	0.64	2.50	51.36
4	20	8.30	3.35	0.24	0.13	0.00	0.00	0.13	0.00	0.44	0.01	0.00	0.08	0.38	0.97	14.05
4	21	37.53	1.09	2.57	0.17	0.03	0.00	1.86	0.08	0.56	0.07	0.02	0.08	0.07	2.34	46.46
4	22	34.57	1.04	3.07	0.17	0.05	0.00	4.17	0.35	0.82	0.07	0.04	0.26	0.14	2.54	47.27
4	23	22.33	0.75	2.11	0.09	0.02	0.00	2.21	0.12	0.61	0.05	0.01	0.12	0.05	1.45	29.91
4	24	27.88	0.85	2.05	0.08	0.02	0.00	1.68	0.07	1.09	0.18	0.01	0.08	0.05	1.95	35.99
4	25	23.37	0.69	2.78	0.14	0.05	0.00	1.90	0.15	0.95	0.09	0.01	0.09	0.05	1.61	31.87

Unofficial
