# Supplementary material for: Determination of Optimal Harvest Time in Cannabis sativa L. Based upon Stigma Color Transition
Source: Plants (Basel). 2025 May 20;14(10):1532. doi: 10.3390/plants14101532 (PMC12114869; doi:10.3390/plants14101532)
Supplement: Supplementary file 1 [file plants-14-01532-s001.zip › Table S2 Inflorescence weight and cannabinoid yield.pdf]

Table S2. The inflorescence weight and cannabinoid yield of all sample inflorescences in the harvest optimization trial (n = 100)

Stage	Plant	Inflorescence Weight (g)	Cannabinoid Yield (g)
1	1	21.4	1.07
1	2	22.8	1.13
1	3	24.4	1.27
1	4	29	1.11
1	5	23.6	0.99
1	6	12.1	0.68
1	7	20.6	1.02
1	8	16.9	0.64
1	9	11	0.50
1	10	8.2	0.03
1	11	16.5	1.30
1	12	8.3	0.18
1	13	40.7	3.08
1	14	30.6	1.48
1	15	14	0.50
1	16	5.8	0.16
1	17	7.4	0.47
1	18	17.2	0.45
1	19	14.9	0.36
1	20	16.3	0.22
1	21	8.3	0.24
1	22	18.6	0.74
1	23	8.4	0.09
1	24	4.4	0.05
1	25	13.5	0.29
2	1	24.6	0.85
2	2	23.5	0.83
2	3	26.8	1.34
2	4	30.5	1.23
2	5	26.5	0.78
2	6	13.8	0.81
2	7	21.3	0.98
2	8	25.9	0.92
2	9	11.3	0.38
2	10	9.1	0.10
2	11	17.5	1.31
2	12	8.4	0.31
2	13	55.6	4.84
2	14	40.3	3.22
2	15	14.7	0.96
2	16	6.6	0.20

Unofficial

2	17	8.9	0.66
2	18	17.4	0.62
2	19	15.5	0.46
2	20	16.5	0.20
2	21	9	0.45
2	22	20	1.04
2	23	8.7	0.16
2	24	5.3	0.22
2	25	15.6	0.28
3	1	26	1.00
3	2	24.4	1.05
3	3	58.4	3.74
3	4	43	1.89
3	5	25	1.03
3	6	14.4	0.85
3	7	22.7	1.22
3	8	39.4	2.90
3	9	11.9	0.52
3	10	15.4	0.55
3	11	14.6	1.06
3	12	8.9	0.31
3	13	65.6	4.84
3	14	46.8	3.99
3	15	27.7	1.65
3	16	7.6	0.30
3	17	21	1.70
3	18	64.4	4.87
3	19	16.4	0.76
3	20	16.6	0.25
3	21	16.9	0.88
3	22	37.1	2.07
3	23	7.4	0.14
3	24	23.3	1.25
3	25	20.7	0.74
4	1	26.4	1.45
4	2	25.2	1.39
4	3	77.3	5.28
4	4	40.3	2.02
4	5	26.3	1.73
4	6	12.4	0.85
4	7	25.9	1.31
4	8	41.9	2.73
4	9	12.4	0.74
4	10	16.3	0.55

4	11	23	1.49
4	12	23.6	1.15
4	13	83.6	6.56
4	14	69.2	5.73
4	15	14.1	0.80
4	16	13.7	0.50
4	17	33.2	2.71
4	18	83.4	6.97
4	19	18.3	0.94
4	20	17.1	0.24
4	21	14.9	0.69
4	22	46.9	2.22
4	23	10.3	0.31
4	24	11.8	0.42
4	25	19.6	0.62
