# Supplementary material for: Determination of Optimal Harvest Time in Cannabis sativa L. Based upon Stigma Color Transition
Source: Plants (Basel). 2025 May 20;14(10):1532. doi: 10.3390/plants14101532 (PMC12114869; doi:10.3390/plants14101532)
Supplement: Supplementary file 1 [file plants-14-01532-s001.zip › Table S4 Harvest days.pdf]

Table S4. The harvest times by day for each inflorescence per cultivar.

Stage	Cultivar No.	Harvest Days	Days since flowering
1	1	Friday, 1 March 2024	31
1	2	Friday, 1 March 2024	31
1	3	Friday, 1 March 2024	31
1	4	Friday, 1 March 2024	31
1	5	Friday, 1 March 2024	31
1	6	Friday, 1 March 2024	31
1	7	Friday, 1 March 2024	31
1	8	Monday, 4 March 2024	34
1	9	Friday, 1 March 2024	31
1	10	Friday, 8 March 2024	38
1	11	Friday, 1 March 2024	32
1	12	Monday, 4 March 2024	34
1	13	Friday, 1 March 2024	32
1	14	Monday, 4 March 2024	34
1	15	Friday, 1 March 2024	32
1	16	Wednesday, 13 March 2024	44
1	17	Friday, 8 March 2024	38
1	18	Friday, 1 March 2024	32
1	19	Friday, 1 March 2024	32
1	20	Friday, 1 March 2024	32
1	21	Friday, 8 March 2024	39
1	22	Monday, 4 March 2024	34
1	23	Monday, 4 March 2024	34
1	24	Wednesday, 13 March 2024	44
1	25	Friday, 8 March 2024	39
2	1	Friday, 8 March 2024	38
2	2	Friday, 8 March 2024	38
2	3	Thursday, 28 March 2024	58
2	4	Friday, 22 March 2024	52
2	5	Wednesday, 13 March 2024	43
2	6	Wednesday, 13 March 2024	43
2	7	Wednesday, 13 March 2024	43
2	8	Friday, 8 March 2024	38
2	9	Friday, 8 March 2024	38
2	10	Monday, 18 March 2024	49
2	11	Monday, 18 March 2024	49
2	12	Wednesday, 13 March 2024	44
2	13	Monday, 18 March 2024	49
2	14	Thursday, 28 March 2024	58
2	15	Monday, 18 March 2024	49
2	16	Monday, 18 March 2024	49
2	17	Monday, 18 March 2024	49
2	18	Wednesday, 13 March 2024	44
2	19	Friday, 8 March 2024	38

Unofficial

2	20	Friday, 8 March 2024	38
2	21	Monday, 18 March 2024	49
2	22	Monday, 18 March 2024	49
2	23	Monday, 18 March 2024	49
2	24	Friday, 22 March 2024	52
2	25	Monday, 18 March 2024	49
3	1	Wednesday, 13 March 2024	43
3	2	Wednesday, 13 March 2024	43
3	3	Tuesday, 28 May 2024	119
3	4	Wednesday, 3 April 2024	64
3	5	Monday, 18 March 2024	48
3	6	Wednesday, 13 March 2024	43
3	7	Monday, 18 March 2024	48
3	8	Monday, 18 March 2024	48
3	9	Wednesday, 13 March 2024	43
3	10	Friday, 22 March 2024	52
3	11	Wednesday, 3 April 2024	64
3	12	Monday, 18 March 2024	49
3	13	Wednesday, 10 April 2024	71
3	14	Monday, 15 April 2024	76
3	15	Monday, 22 April 2024	83
3	16	Friday, 22 March 2024	52
3	17	Wednesday, 3 April 2024	64
3	18	Monday, 13 May 2024	104
3	19	Wednesday, 13 March 2024	44
3	20	Wednesday, 13 March 2024	44
3	21	Wednesday, 3 April 2024	64
3	22	Wednesday, 3 April 2024	64
3	23	Friday, 22 March 2024	52
3	24	Monday, 15 April 2024	76
3	25	Wednesday, 3 April 2024	64
4	1	Thursday, 28 March 2024	58
4	2	Thursday, 28 March 2024	58
4	3	Tuesday, 28 May 2024	119
4	4	Monday, 15 April 2024	76
4	5	Thursday, 28 March 2024	58
4	6	Wednesday, 13 March 2024	43
4	7	Friday, 22 March 2024	52
4	8	Monday, 15 April 2024	76
4	9	Friday, 22 March 2024	52
4	10	Thursday, 28 March 2024	58
4	11	Monday, 29 April 2024	91
4	12	Monday, 29 April 2024	91
4	13	Monday, 22 April 2024	83
4	14	Monday, 29 April 2024	91
4	15	Monday, 29 April 2024	90

4	16	Monday, 29 April 2024	90
4	17	Monday, 22 April 2024	83
4	18	Tuesday, 28 May 2024	119
4	19	Friday, 22 March 2024	52
4	20	Monday, 18 March 2024	49
4	21	Monday, 13 May 2024	104
4	22	Monday, 13 May 2024	104
4	23	Wednesday, 10 April 2024	71
4	24	Monday, 22 April 2024	83
4	25	Monday, 22 April 2024	83
