# Supplementary material for: Determination of Optimal Harvest Time in Cannabis sativa L. Based upon Stigma Color Transition
Source: Plants (Basel). 2025 May 20;14(10):1532. doi: 10.3390/plants14101532 (PMC12114869; doi:10.3390/plants14101532)
Supplement: Supplementary file 1 [file plants-14-01532-s001.zip › Table S5 The mean and median harvest day of each amber stage.pdf]

Table S5. The mean and median harvest days for each amber stage.

Stage	Mean (Days)	Median (days)
1	34	32
2	46	49
3	61	52
4	77	83

Unofficial
